# Testing the effectiveness and acceptability of online supportive supervision for mental health practitioners in humanitarian settings: a study protocol for the caring for carers project

**DOI:** 10.1186/s12888-023-05246-1

**Published:** 2023-11-28

**Authors:** Ruth Wells, Ceren Acarturk, Muhammad Kamruzzaman Mozumder, Gülşah Kurt, Louis Klein, Salah Addin Lekkeh, Ammar Beetar, Sabiha Jahan, Fatema Almeamari, Md. Omar Faruk, Michael McGrath, Syeda Fatema Alam, Mustafa Alokoud, Ranak Dewan, Ahmed El Vecih, Hafsa El-Dardery, Dusan Hadzi-Pavlovic, Hanan Hammadi, Mounir Al Shekh Hamoud, M. Tasdik Hasan, Rohina Joshi, Sowmic Kothaa, Fauzia Kabir Chowdhury Lamia, Chiara Mastrogiovanni, Hussam Najjar, Shaun Nemorin, Kathryn Nicholson-Perry, Tahmina Sarker Prokrity, Rania Said Yousef, Mamoun Tawakol, Ersin Uygun, Wael Yasaki, Scarlett Wong, Ariel Zarate, Zachary Steel, Simon Rosenbaum

**Affiliations:** 1 School of Clinical Medicine, Discipline of Psychiatry and Mental Health, University of New South Wales, Sydney, Australia; 2 Department of Psychology, Koc University, Istanbul, Türkiye; 3 Department of Clinical Psychology, University of Dhaka, Dhaka, Bangladesh; 4 Hope Revival Organization, Gaziantep, Türkiye; 5 Action Lab, Department of Human Centred Computing, Faculty of Information Technology, Monash University, Melbourne, Australia; 6 School of Population Health, University of New South Wales, Sydney, Australia; 7 New South Wales Service for the Treatment and Rehabilitation of Torture and Trauma Survivors (STARTTS), Sydney, Australia; 8 Discipline of Psychological Science, Australian College of Applied Psychology, Sydney, Australia; 9 Trauma and Disaster Mental Health, Bilgi University, Istanbul, Türkiye; 10 Suicide Prevention Subgroup, Cox’s Bazar, Bangladesh

**Keywords:** Online clinical supervision, Mental health practitioners, Humanitarian settings

## Abstract

**Background:**

Local humanitarian workers in low and middle-income countries must often contend with potentially morally injurious situations, often with limited resources. This creates barriers to providing sustainable mental health and psychosocial support (MHPSS) to displaced individuals. Clinical supervision is an often neglected part of ensuring high-quality, sustainable care. The Caring for Carers (C4C) project aims to test the effectiveness and acceptability of online group-based supportive supervision on the well-being of MHPSS practitioners, as well as service-user-reported service satisfaction and quality when working with displaced communities in Türkiye, Syria, and Bangladesh. This protocol paper describes the aim, design, and methodology of the C4C project.

**Method:**

A quasi-experimental, mixed-method, community-based participatory research study will be conducted to test the effectiveness of online group-based supportive clinical supervision provided to 50 Syrian and 50 Bangladeshi MHPSS practitioners working with Syrian and Rohingya displaced communities. Monthly data will be collected from the practitioners and their beneficiaries during the active control (six months) and supervision period (16 months over two terms). Outcomes are psychological distress (Kessler-6), burnout (the Copenhagen Burnout Inventory), compassion fatigue, compassion satisfaction, and secondary traumatic stress (Professional Quality of Life Scale), perceived injustice, clinical self-efficacy (Counseling Activity Self-Efficacy Scale), service satisfaction, and quality (Client Satisfaction Questionnaire and an 18-item measure developed in this project). A realist evaluation framework will be used to elucidate the contextual factors, mechanisms, and outcomes of the supervision intervention.

**Discussion:**

There is a scarcity of evidence on the role of clinical supervision in improving the well-being of MHPSS practitioners and the quality of service they provide to displaced people. By combining qualitative and quantitative data collection, the C4C project will address the long-standing question of the effectiveness and acceptability of clinical supervision in humanitarian settings.

**Supplementary Information:**

The online version contains supplementary material available at 10.1186/s12888-023-05246-1.

## Introduction

The number of people who have been forcibly displaced due to ongoing conflicts, violence, and persecution is currently at an all-time high. Displaced individuals are at a higher risk of experiencing mental health problems and functional impairment as a result of the various stressors they face before, during, and after displacement [1]. The majority of displaced people live in low- and middle-income countries (LMICs), where access to mental health treatment is limited [2]. The overwhelming need for mental health services in LMICs poses a challenge for already stretched health systems, which often lack the infrastructure and resources to provide sustainable, culturally appropriate mental health and psychosocial support (MHPSS) [2]. Additionally, there is a shortage of mental health professionals in these countries, making it difficult to deliver specialized treatments. Therefore low-intensity, intercultural, and scalable psychosocial interventions based on task-sharing have emerged as a potential solution to address the mental health needs of forcibly displaced individuals in low- and middle-income countries.

One novel approach to addressing the burden of needs in low resource contexts is called ‘task-sharing’ in which tasks typically performed by specialized mental health professionals are transferred or shared with individuals who have little or no formal mental health education, such as community health workers. These individuals receive brief training in order to provide mental health care [3]. Clinical supervision from mental health experts is crucial to support the quality of care provided by these workers and the sustainability of psychosocial services [4].

Supportive clinical supervision is a collaborative, emotional, and practical professional support that can help practitioners cope with the stress of working in displacement contexts. It is a vital aspect of mental health practice, providing practitioners with the support they need to improve their skills, maintain quality of care, and ensure the sustainability of psychosocial services [5]. By reducing staff turnover and increasing job satisfaction and motivation, supervision can play a critical role in protecting practitioners from burnout and mental health problems, such as depression, anxiety, and post-traumatic stress symptoms [6–10]. Despite its importance, supervision is often overlooked in humanitarian mental health programming due to resource constraints [11, 12].

In addition, there is a well-established link between contextual stressors such as occupational stress, structural injustice and high workloads and negative psychological outcomes such as distress and burnout. There has been little research to date on how to protect practitioners involved in task-sharing from these negative psychological outcomes.

To ensure that practitioners can provide high-quality, sustainable care, it is essential to prioritize supervision in displacement contexts. Furthermore, there is a lack of research investigating the impact of supervision on the mental health outcomes of displaced communities who use psychosocial services [13]. Despite the potential benefit to the practitioners, organizations and the service user, the acceptability and effectiveness of clinical supervision for mental health practitioners in displacement contexts has not been extensively studied.

The aim of the Caring for Carers (C4C) project is to evaluate the impact of online supportive clinical supervision on the well-being of mental health and psychosocial support (MHPSS) practitioners, as well as service-user reported service satisfaction and quality, when working with Syrian and Rohingya displaced communities in three prolonged displacement contexts: Türkiye, Syria, and Bangladesh. By considering the perspectives of various stakeholders, including mental health practitioners, Syrian and Rohingya mental health service users, supervisors, and organizations, the project also aims to assess the acceptability and appropriateness of the supervision program.

## Study objectives

Drawing on a realist evaluation framework [14]; we aim to evaluate the effectiveness of the online clinical supervision program both in terms of process and outcomes. The overall objectives of the C4C project are:

*Objective 1*: Characterise the relationship between contextual stressors (post-migration living difficulties, perceived injustice, and trauma events) and psychological outcomes (psychological distress (K6) and PTSD (PTSD8) and burnout.

### Hypothesis 1

During the active control period, contextual stressors will be positively associated with psychological outcomes among MHPSS practitioners working with displaced communities, both at baseline and longitudinally. Compassion satisfaction will mediate the relationship between contextual stressors and psychological outcomes.

*Objective 2*: To evaluate whether the 16-month online supervision program ameliorates the negative impact of contextual stressors on psychological outcomes.

### Hypothesis 2

The negative relationship between contextual stressors and psychological outcomes during the intervention period will be weaker, compared to the active control period,

### Hypothesis 3

Practitioner clinical self-efficacy will increase at a greater rate during the intervention period compared the active control period, controlling for contextual stressors and psychological outcomes,

*Objective 2*: To evaluate the effectiveness of the 16-month online supervision program (2 8-months terms) on perceived service satisfaction, acceptability, and appropriateness of the service provided to Syrian and Rohingya displaced communities in Türkiye, Syria, and Bangladesh compared to a 6-month active control period.

*Objective 3*: To identify supervision group content and processes associated with greater improvements in practitioner wellbeing and service user satisfaction.

## Conceptual framework

The current project is a mixed-method, longitudinal, quasi-experimental, and community-based participatory research study using a realist evaluation framework. This design is feasible in a low-resource setting and does not require withholding the intervention from anyone.

## Realist evaluation framework

The realist evaluation (RE) framework is a theory-driven approach that aims to uncover how, for whom, and under what circumstances an intervention, or a program works [14]. This approach acknowledges the complexities of evaluating health interventions as the observed outcome is inextricably linked with contextual factors. Combining routine qualitative and quantitative data collection and analysis, the RE framework examines processes and mechanisms underpinning the intervention [15, 16]. RE-guided research inquiry starts with an initial program theory (IPT) that postulates how, why, for whom, and under what conditions an intervention works based on previous knowledge. It employs context-mechanism-outcome (C-M-O) configuration as a primary analytical tool to delineate how specific contextual factors activate certain mechanisms, leading to the intervention outcome. During the data collection and analysis, C-M-Os are iteratively tested, specified, and integrated into the IPT to produce a refined middle-range theory of how an intervention works in a specific context. This approach is particularly useful for policymakers and program designers as it provides practical guidance about the effectiveness, transferability, and adaptability of an intervention in different contexts [15].

**Fig. 1:**
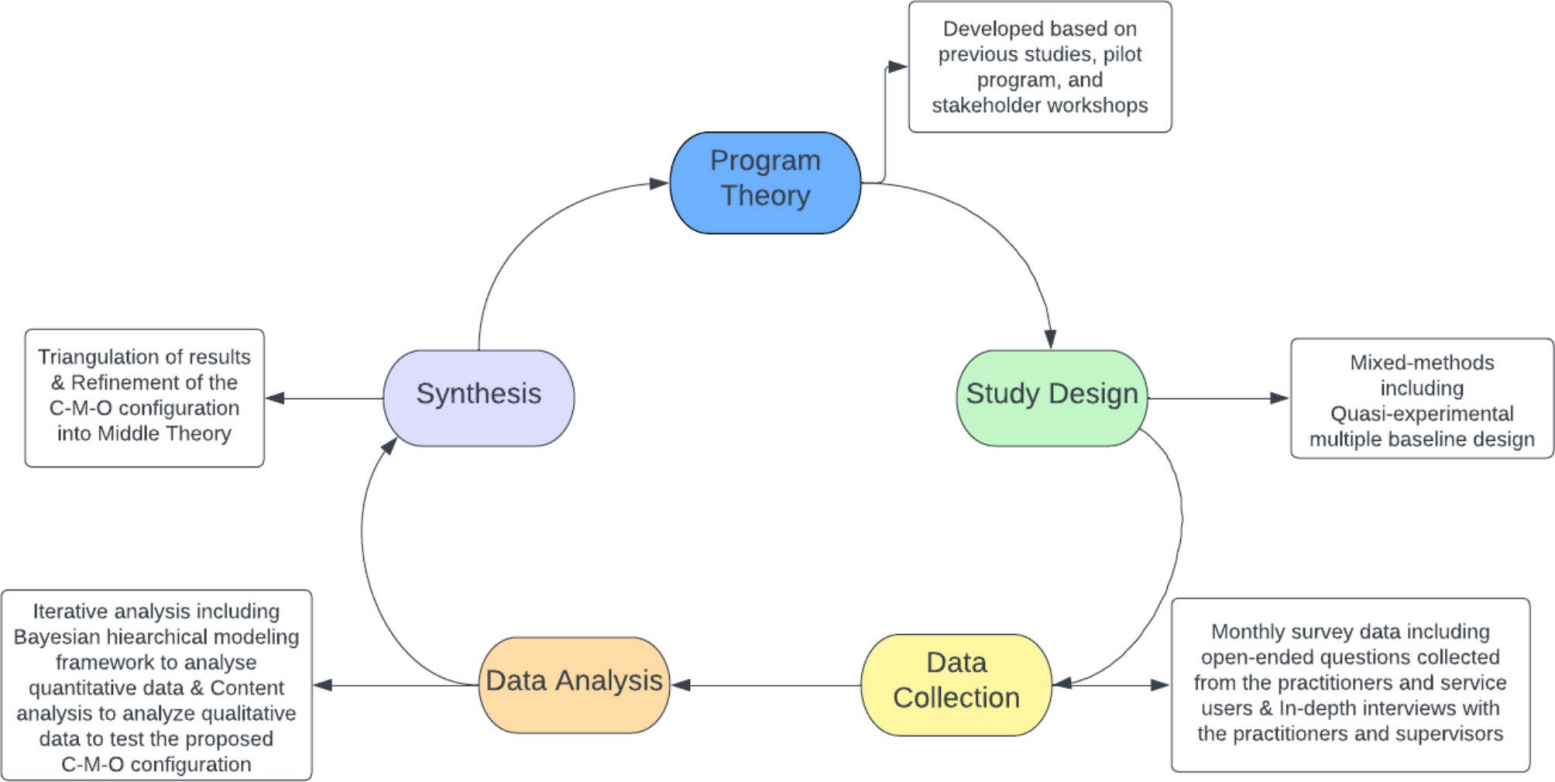
Realist Evaluation Process and Cycle

**Fig. 2:**
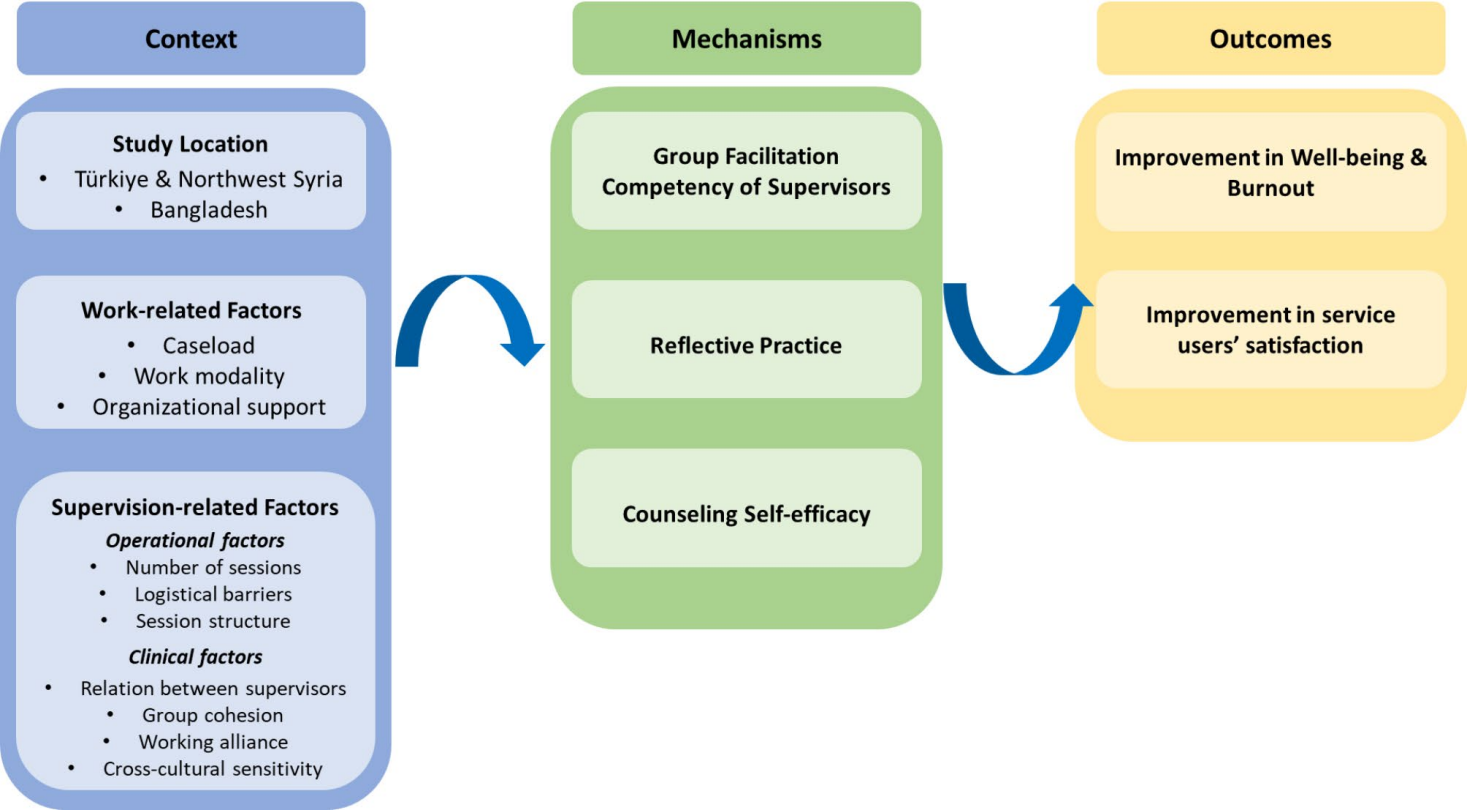
The C-M-O Configuration

The application of the RE framework in the scope of this project is depicted in Fig. 1. The C-M-O configuration is given in Fig. 2. We developed our IPT based on the previous findings on supportive supervision [12], our pilot program findings, and stakeholder workshops aimed to identify the intended outcomes, enablers, and barriers of the supervision program. Each component of the IPT is explained below.

### Contextual factors

The contextual factors are given under three categories: (1) Study Location; (2) Work-related Factors; and (3) Supervision Program-related Factors.

### Study context

Conducting psychosocial work with communities who have experienced conflict, persecution and displacement can increase the risks of experiencing moral injury and burnout [17]. This is particularly the case in contexts where resources to address communities needs are scarce.

### Türkiye and Northwest Syria

The Syrian crisis led to the displacement of 13.3 million Syrians, of which 6.6 million were forced to leave the country and 6.7 were internally displaced [18]. Many of those internally displaced persons (IDPs) live in dire conditions in the camps in Northwest Syria. They live under constant fear with minimal or no access to basic needs such as proper housing, hygiene conditions, and food. Therefore, IDPs are at heightened risk of mental health difficulties due to conflict and daily living hardships [19]. Ongoing bombings and attacks in the region eroded the public health system, forced healthcare professionals to leave the country, and thereby worsened health conditions among IDPs [20]. The lack of sufficient MHPSS practitioners to provide psychosocial support put pressure on these practitioners and is not sufficient to meet the need [21].

Among Syrians who sought refuge in other countries, the majority or roughly 3.6 million people, live in Türkiye under temporary protection status. Türkiye limits refugee status to those escaping from European countries; therefore, Syrians are given temporary protection status, which provides limited access to basic services including health, education, and employment [22]. In addition to conflict-related traumatic experiences, they must contend with a multitude of resettlement-related stressors, such as financial difficulties, language barriers, access to stable internet connection, and social isolation. Due to these difficulties, they are at high risk of mental health problems [23]. Although they can utilize mental health services, only a minority seek professional psychological help, indicating a major treatment gap in the community [24].

### Bangladeshi

Rohingya people have a long history of human rights violations, oppression, and persecution. They became stateless following the Citizenship Act of 1982, which denied citizenship to the Rohingya people in Myanmar [25]. Approximately 30,000 Rohingya people have lived in displacement in Cox’s Bazaar in Bangladesh since 1992. In 2017, Myanmar’s military operation of ethnic cleansing toward the Rohingya people led to the unpreceded exodus of 750 000 people to Cox’s Bazar in Bangladesh. Currently, around one million Rohingya people live in Cox’s Bazar, the largest refugee camp in the world [26]. As Bangladesh is a resource constrained country and not a party to the 1951 Geneva Convention or its 1967 protocol, Rohingya people face substantial challenges related to international protection. They are confined to camps, which exacerbates the adversities caused by decades-long human rights violations. They lack freedom of movement and access to basic services such as education and employment in Bangladesh. Uncertainty about the future, safety concerns, and camp conditions with limited access to stable internet connection compounds existing vulnerabilities, thereby substantially increasing the risk of mental health problems [27, 28]. Although many national and international organizations provide MHPSS services to Rohingya people, the provision of culturally appropriate psychosocial support is limited [28] and MHPSS service utilization is minimal [29].

### Work-related factors

Working in direct contact with people who have experienced significant adversity in resource constrained environments can pose psychological and social hazards to humanitarian workers. Work conditions represent the main proximal context factors that can hinder or promote supervision [8]. Excessive workload, lack of organizational support, lack of time and space allocated for supervision, logistical barriers, and geographical location hampers prioritizing supervision in displacement contexts [30] and impedes practitioners’ capacity to benefit from supervision programs [8]. These conditions are also the main determinants of the mental health of humanitarian workers [31, 32].

Humanitarian workers providing support to displaced people are at higher risk of a wide range of mental health problems. Recent research has shown that at least one-third of MHPSS practitioners working with Syrian refugees in Türkiye and the Syrian border are likely to experience depression, anxiety, burnout, and secondary traumatization due to the stressful nature of their jobs [33]. The risk of experiencing mental health problems is further elevated among practitioners from the displaced community as previous trauma exposure increases the vulnerability to work-related distress [32]. Furthermore deteriorated working conditions during COVID-19 adversely impacted the mental health of humanitarian workers in Bangladesh [34], Syria and Türkiye [35].

Thus, in the present study, we will examine work conditions (e.g., caseload, work modality, and organizational support) to understand the enablers and barriers of our supervision program.

### Supervision-related factors

In addition to contextual factors, we hypothesize that supervision-related factors will impact on outcomes. This includes operational supervision factors, such as the number of supervision sessions attended and session structure (e.g., time allocated for different activities during the session) are likely to impact the outcome of the supervision program. Clinical supervision factors could include the working relationship between the co-supervisors; group cohesion; working alliance between the supervisees and supervisors; cultural sensitivity of the group members; disparities in relative experience and clinical frameworks among group members.

### Mechanisms

Within the RE framework, ‘mechanisms’ refer to the underlying processes whereby the intervention results in the observed outcome. An intervention triggers specific mechanisms/processes in a particular context, thereby producing the outcome [14]. We propose three key mechanisms underpinning the supervision outcome: 1) group facilitation competencies of supervisors; and 2) reflective practice as the central pillar of the supervision program 3) the counseling self-efficacy of MHPSS practitioners. Counseling self-efficacy refers to practitioners’ belief in their ability to perform counseling tasks and activities [36]. A recent systematic review showed that clinical supervision enhances the efficacy of practitioners on counseling skills, which then improves mental health and well-being [37]. Counseling self-efficacy, in turn, is associated with supervision programme effectiveness [38]. Further, the competencies of supervisors play a vital role in providing ethical and quality clinical supervision [39]. Those competencies refer to the skills and knowledge of the supervisors required to provide supervision [40]. Depending on the modality of supervision, required competencies might vary.

As the current project focuses on group supervision, competencies of the supervisors related to group facilitation (e.g., establishing group guidelines and/or ground rules, fostering empathy between group members, collaborative problem solving [41]) will be investigated as a potential mechanism. Finally, reflective practice or reflection can be defined as deliberate thinking about knowledge, skills, and experiences to develop a new perspective or understanding in practice [42]. It involves exploring actions, experiences, and feelings and drawing connections between learnings from this process and their impacts on practitioners and clients. As a main component of supervision, teaching and fostering reflective practice is key to enhancing the counseling skills of practitioners [43] and promoting quality service [44]. As such, we assume that reflective practice will be a mechanism behind intendent positive outcomes of this project. We are also aware that reflective practice may not be culturally or contextually appropriate, so other factors, such as skill acquisition may emerge as mechanisms.

### Outcomes

As the last element of the C-M-O configuration, we propose the following outcomes for our supervision program:

1. The relationship between psychological hazards presented by the context and work -related factors will be ameliorated by the introduction of clinical supervision. That is, relative to adversities experienced, practitioners will show reduced distress, PTSD and burnout during the supervision program compared to during the active control period.
2. Improvement in MHPSS service satisfaction of Syrian and Rohingya displaced communities relative to the active control period.

RE framework entails an iterative process of testing C-M-O configurations and refining the initial theory by collecting and analysing empirical data [14]. To better understand local context and needs, involving multiple stakeholders in this process is highly encouraged [45]. In this project, this will be achieved by embracing the community-based participatory research (CBPR) approach, which aims to establish a cooperative and trusting relationship based on mutual learning, exchange, and equitable representation between communities, researchers, and practitioners in the research projects [46]. This approach is key to ensuring the cultural and ethical conduct of research, which can maximize the benefit, impact, and outreach of the intervention within and across communities [47]. In the current project, multiple stakeholders- researchers, practitioners, supervisors, organizations, and displaced communities- will be included in the research design, implementation, and dissemination to test the proposed links and refine the initial theory around supervision in displacement contexts.

## Study methodology

### Study design and intervention

A quasi-experimental multiple-baseline design will compare repeated measurements of the same individual during an active control period to those during the intervention, eliminating the need for a control group [48]. During the active control component, participants will be provided with freely available MHPSS focused resources, collated in partnership with our stakeholders. During the intervention component, MHPSS practitioners in Syria, Türkiye, and Bangladesh will participate in fortnightly 90-minute supervision sessions facilitated by two co-supervisors; one international and one local psychologist, counseling social worker, or counselor.

MHPSS practitioners will be asked to complete online surveys during the active control component and intervention component to examine the effect of the supervision program on the well-being, burnout level, and counseling self-efficacy of MHPSS practitioners (Objectives 1 & 2). MHPSS service users will be interviewed by trained research assistants to rate service satisfaction and quality (Objective 3).

#### Supervision intervention

The intervention was designed by an Australian clinical psychologist and clinical supervisor with experience providing supervision in both Australia and in humanitarian contexts (SW). The program aims to develop key competencies in mental health practice based on professional practice standards outlined in Supplementary material 1. As the supervision process draws heavily on Western, English-language approaches to supervision, the intervention was adapted in collaboration with local psychologists and psychiatrists (AB, SL, MKM, SJ, OF) following a pilot program [see [49]].

The program uses Australian mental health professionals with specialized training in supervision, along with local supervisors who have professional experience and cultural insight. Supervision will be in groups of 4–6 supervisees, 2 co-supervisors (one Australian and one local supervisor), and a research assistant. Sessions will run for 90 min on Zoom, fortnightly, for 16 months, split into two terms as per postgraduate mental health programs. Groups will be closed once they start, to allow for cohesion and safety. At the end of the first term, participants will be reassigned to a different group with new supervisors and co-supervisees. Supervisor dyads are then placed into a Whatsapp group with a Research Assistant/translator two weeks prior to the supervision groups commencing and will be encouraged to begin a dialogue. The supervisors will be provided with the written guidance and asked to discuss and share ideas with each other.

The supervision models built on reflective and supportive supervision. Reflective supervision helps supervisors guide supervisees to better understand clinical issues. It involves two-way communication and draws on the supervisee’s expertise. This approach is suitable for cross-cultural programs where Australian supervisors may not understand cultural and contextual dynamics. The program also draws on the Integrated Model for Supervision by the International Federation of Red Cross and Red Crescent Societies (IFRC) [12]. Supervisors attend two preparatory workshops and regular reflective group supervision sessions to support problem-solving in a transcultural context. Sessions are designed to focus on case presentations, which are a common format for supervision [50–52]. The program takes a flexible, needs-based approach to supervision, given the varied backgrounds of supervisees and supervisors, as well as the unique cross-cultural, cross-discipline, online, and co-supervision factors. Given that there approximately 52 models of clinical supervision, many with limited research support [53], supervisors are encouraged to apply their preferred models based on the needs of supervisees in each session. While not prescribing a specific model of supervision, the program offers readings and training on various supervision models, as well as a handbook with contextual information and suggestions for structure and process.

### Participants and recruitment

#### Study population

The total sample size for the project is 2,300 comprised of the following samples from each of the participant groups:

1. Participant Group 1 MHPSS Clinicians: 100 (2300 within-subject measurements; 23 monthly per clinician). Participant Group 1 will be equally split between the two data collection sites i.e. 50 practitioners sampled from Türkiye and Northwest Syria, and 50 practitioners sampled from Bangladesh.
2. Participant Group 2 Beneficiaries: 2,200 (between-subjects; 22 monthly per clinician).

This sample size is sufficient to meet the research aims and answer the research questions because in longitudinal growth modelling, sample size is calculated based on the number of assessment occasions and does not require large numbers of participants to achieve sufficient statistical power. We have previously conducted Monte Carlo simulations using the same primary outcome to determine that a sample size of 80 is sufficient to achieve a power of 80% with a similar multiple baseline design with 10 measurement occasions [54]. We have oversampled by 20% given the power calculation of 80 because we expect at least 20% attrition in the unpredictable study locations.

#### Recruitment strategy

MHPSS practitioners will be recruited via the network of the project partners (Hope Revival Organization (HRO) in Türkiye/Syria; Suicide Prevention Sub-Group (SPSG) of the MHPSS Working Group in Bangladesh). MHPSS organisations will be invited to participate in the study. Upon approval to participate in the study, those organisations will be asked to provide a list of consenting MHPSS practitioners in their organisations who have indicated interest in the study. The research team will oversee the recruitment of the practitioners. Recruitment will be open to new participants during the 6-month baseline period and cease once the first term of the supervision program starts. If appropriate (others have dropped out and new practitioners have joined the organisation), new practitioners may join in the break between the two supervision terms when new groups are formed.

#### Inclusion criteria

Inclusion criteria for the *MHPSS practitioners* are: (1) 18 years or over; (2) self-identify as Syrian or Bangladeshi; (3) working as an MHPSS practitioner (psychosocial worker, psychologist, psychiatrist, case worker or psychological counsellor) with displaced Syrian (in Northwest Syria or Türkiye) or Rohingya community (in Bangladesh). Rohingya MHPSS practitioners cannot be included in the study due to Bangladesh Telecommunication Regulatory Commission restrictions on internet access for Rohingya living in Cox’s Bazaar refugee camps since 2019 [55].

Inclusion criteria for *MHPSS service* users are: (1) 18 years or over; and (2) receiving MHPSS services from an MHPSS practitioner recruited in the study. MHPSS service users will be recruited among the beneficiaries of the practitioners involved in the study.

Inclusion criteria for Australian supervisors are: (1) 18 years or older; (2) psychologists, clinical psychologists, social workers or counsellors; (3) completed tertiary training in clinical psychology, social work or counselling or Registered Psychologists.

Inclusion criteria for local supervisors are: (1) Completed a university degree in psychological counselling, psychology or psychiatry; and, (2) to have participated in the pilot supervision program since the beginning of 2020 or have other supervision experience.

#### Remuneration

Syrian and Bangladeshi supervisors and clinicians will be offered two free online short courses to support their participation in the program. Upon completion, they will receive two accredited certificates and digital badges stating that they have completed two short courses at the University of New South Wales Faculty of Medicine and Health, Sydney, Australia. These two certificates confirm participation in a 16-month supervision program.

For practitioners participating in the supervision program, the five people who answer the highest number of questionnaires closest to the date they are sent out will be awarded $50 for each supervision term.

### Measures

#### Practitioner online surveys

The Kessler-6 [56] ), a 6-item measure of general distress which is sensitive to change during treatment; The Copenhagen Burnout Inventory (CBI) [57] 19 item self-report measure with personal, work-related and client-related burnout sub-scales; The Professional Quality of Life (Stamm, 2005), 30 items assessing clinician compassion satisfaction, compassion fatigue and secondary traumatic stress (symptoms of posttraumatic stress disorder associated with helping populations that have experienced trauma). Counseling Activity Self-Efficacy Scales (CASES) [36] a self-rating scale for counselling clinicians to rate their confidence in providing effective counselling; The PTSD-8 [58], a brief measure of PTS symptoms which has been derived from the Harvard Trauma Questionnaire, along with a list of Traumatic Events (HTQ-TEs) Inventory [59]; modified version of Post-migration Living Difficulties (PMLD-17) Questionnaire [60, 61]. Subjective experiences of supervision were measured with the six-item Perceived Supervision Scale (PSS) [62]. A shortened, six-item version of the Turnover Intention Scale (TIS-6) [63] will measure MHPSS practitioners’ intention to leave their current employment. Nine questions adapted from the Demographic and Health Survey Service Provision Assessment [64], will capture organizational and workforce characteristics.

#### Beneficiary interviews

Service satisfaction and quality among MHPSS service users will be measured by the Client Satisfaction Questionnaire (CSQ-8) [65] and an 18-item measure developed in this project to evaluate displacement context-specific MHPSS service use experiences.

The data collection plan with an overview of measures for each participant group is given in Table 1 for the active control period and Table 2 for the intervention period.

**Table 1:** Timeline for Active Control Period

Measures	Baseline	1st month	2nd month	3rd month	4th month	5th month	6th month
Demographics	x						
HTQ-TEs*	x						
Location details	x	x	x	x	x	x	x
Kessler-6	x	x	x	x	x	x	x
ProQOL-19	x	x	x	x	x	x	x
CBI	x	x	x	x	x	x	x
Injustice	x		x		x		x
PMLD-17*	x		x		x		x
CASES	x				x		x
PTSD-8	x						x

*Only included in the Syria/Türkiye site

**Table 2:** Timeline for Intervention Period

Measures	1st month	2nd month	3rd month	4th month	5th month	6th month	7th month	8th month
Organizational survey	x							
Location details	x	x	x	x	x	x	x	x
Kessler-6	x	x	x	x	x	x	x	x
ProQOL-19	x	x	x	x	x	x	x	x
CBI	x	x	x	x	x	x	x	x
Injustice		x		x		x		x
PMLD-17*		x		x		x		x
CASES				x				x
PTSD-8								x

*Only included in Türkiye/Syria site

### Procedures

#### Practitioner online surveys

Each month, field research teams for each site contact all currently eligible practitioners (WhatsApp and email) and provide them with an online survey link containing all planned measures for that cross-section of the overall program. Surveys are delivered using the online KoBoToolbox platform [66]. KoBoToolbox was selected over alternatives (for e.g., REDCap) due to its offline data collection and multilingual support.

#### Beneficiary interviews

Practitioners who deliver MHPSS services directly to beneficiaries are eligible for enrolment in our beneficiary interview data collection program. Note, not all practitioners enrolled in our supervision program are eligible for beneficiary interviews for a range of circumstances. Agreements with the MHPSS service organisations are required to contact beneficiaries, with some organisations not able to agree to this process, other reasons for not collecting beneficiary data include: practitioner has changed jobs into a non-service delivery role (i.e. line manager/supervisor); fractional unemployment; as well as illness or holiday. For each eligible practitioner, field researchers at each site will attempt to conduct an interview with one of the beneficiaries of their MHPSS services, on a one-to-one basis per interview cycle (see Table *Beneficiary data collection* for description of interview cycles). Beneficiaries are eligible for an interview up to 21 days after their session with their MHPSS practitioner.

Practitioners are blinded from knowing which of their beneficiaries receive an interview; except in such cases where only a single beneficiary is available for interview per interview cycle. Beneficiaries were randomly selected from among all beneficiaries seen by the practitioner in a given week based on the time at which the session with the practitioner occurred to minimize day-of-week and time-of-day sampling biases [67, 68]. A novel sampling procedure was developed to counter-balance across available time windows (see Supplementary Material 2).

#### Supervision program participation

The proposed online supervision program for practitioners in will be conducted over 16 months, divided into two 8-month terms, and co-facilitated by an international and a local supervisor. To ensure feasibility and cost-effectiveness in displacement contexts [5], group supervision sessions for 4 to 6 practitioners will be held fortnightly for 90 min on the Zoom platform. In-country research assistants (referred to as “field researchers”) will coordinate meeting invitations and hosting and attend each session. They will also seek consent to record the sessions and remind practitioners two days before their scheduled sessions while monitoring their attendance.

### Analytic design

#### Quantitative data

Hierarchical models will be used to compare practitioner rate of change in reported outcomes between the Active control period and each of the Supervision Terms 1 and 2.

As such, prospective models will consider the Active Control data as a within-subjects control condition that can be jointly estimated across levels of the model (i.e., practitioner, supervision group, site). Cross-sectional data drawn from the beneficiary interviews will be nested within practitioner from the longitudinal data drawn from the online practitioner surveys, cross-classified across time. Supervision program participation data may also be incorporated following qualitative analysis.

To address Objective 1, data from the active control period will be used to model the relationships between psychological hazards and outcomes, using the hierarchical model structure described above. This model will take into account sociodemographic characteristics, organizational factors, group allocation, exposure to the intervention, and other contextual factors that may be identified over the course of the intervention program. To address Objective 2, the same model will be applied to data from the supervision terms to determine whether the introduction of supervision moderates the relationships between psychological hazards and outcomes identified in Objective 1.

Data may be transformed and/or combined in order to achieve appropriate variance partitioning (for e.g., factor analytic techniques, clustering), informed by gold-standard approaches [69]. All candidate variables will be visualized and modelled at the bivariate level prior to final analysis in order to mitigate multicollinearity during model fitting; as such, not all planned variables may be suitable for inclusion in finalized models. This iterative exploratory process means that models cannot be specified in advance of data collection; analyses will therefore be pre-registered, where possible, to ensure best practise [70].

Planned statistical analyses will primarily be carried out in the R language ecosystem [71] within the RStudio IDE [72], however, MPlus [73], STATA [74], and SPSS [75] may also be utilized. Data collection will be conducted primarily using platforms such as KoBoToolbox [66] and Qualtrics [76].

Planned reporting will be performed in accordance with Strengthening the Reporting of Observational Studies in Epidemiology (STROBE) guidelines [77].

#### Qualitative data

Video recordings of the supervision sessions will be analysed using content analysis [78] to identify both process (how do things happen in the session) and content (what is being discussed in the session) codes. Content codes will be iteratively devised in collaboration with researchers across all sites to promote the cultural and contextual relevance to codes. Qualitative analysis of the videos will help us to gain insights into the supervision process and examine the proposed mechanisms. Information on the number of supervision sessions attended, logistical barriers (e.g., connection issues), and session structure will also be extracted from the video recordings.

Thematic analysis [79, 80] will be conducted on a subset of supervision videos from the beginning, middle, and end of the supervision program to elucidate proposed mechanisms (supervisor practices; group processes; barriers and facilitators to participation). Further, at the start and end of each supervision term, semi-structured interviews will be conducted with the practitioners to gain insights into the impact, acceptability, and appropriateness of the supervision program. NVivo 12 software will be used to aid qualitative data analysis [81].

## Discussion

Clinical supervision has been identified by international consensus as a key global research priority for promoting quality mental health care for displaced communities [82]. Clinical supervision is conducive to the well-being and skills improvement of MHPSS practitioners as well as the satisfaction of service users [12, 13]. However, evidence on its effectiveness in displacement settings is lacking. The Caring for Carers (C4C) project aims to provide and test the acceptability and effectiveness of a culturally tailored and feasible online group-based clinical supervision program for MHPSS practitioners and service users in Türkiye, Syria, and Bangladesh. Based on the Realist Evaluation Framework, the project involves routine qualitative and quantitative data collection and active involvement of multiple stakeholders in the program’s design, delivery, and dissemination of outcomes. The project outcomes can guide the transfer and adaptation of the program to other displacement contexts.

### Electronic supplementary material

Below is the link to the electronic supplementary material.

Supplementary Material 1: Supervision Resources and Framework.

Supplementary Material 2: C4C Project R Workbook Setup.

## Data Availability

Not applicable.
