# Supplementary material for: Testing the effectiveness and acceptability of online supportive supervision for mental health practitioners in humanitarian settings: a study protocol for the caring for carers project
Source: BMC Psychiatry. 2023 Nov 28;23:884. doi: 10.1186/s12888-023-05246-1 (PMC10683137; doi:10.1186/s12888-023-05246-1)
Supplement: Supplementary file 1 — Supplementary Material 1: Supervision Resources and Framework. [file 12888_2023_5246_MOESM1_ESM.docx]

Supplementary File 1

Supervision Materials

As described in the Integrated Model for Supervision created by the International Federation of Red Cross and Red Crescent Societies, “Supervision is a safe, supportive, confidential and collaborative relationship between a supervisor and/ or supervisee(s), where supervisees can voice their difficulties, discuss mistakes and be recognized for their successes, receive constructive feedback and emotional support, and build their technical skills and capacity [1].

The model that Australian institutions now predominantly disseminate is based on Competency-Based models of supervision [2]. The program was design to improve key competencies outlined in international psychology professional practice guidelines (IPCP; AHPRA) and humanitarian organisation practice guidelines (IFRC; MSF; GIZ) [3–5], World Health Organisation guidelines (WHO EQUIP and MHGap) and the Interagency Standing Committee Guidelines (IASC) [6] . The key competencies are outlined below.

All supervisors were provided with a handbook to guide their involvement in the program, which is further provided below.

**References**

1. Perera C, McBride KA, Travers Á, Tingsted Blum P, Wiedemann N, Dinesen C, et al. Towards an integrated model for supervision for mental health and psychosocial support in humanitarian emergencies: A qualitative study. PLoS ONE. 2021;16:e0256077.

2. Gonsalvez CJ, Calvert FL. Competency-based Models of Supervision: Principles and Applications, Promises and Challenges. Australian Psychologist. 2014;49:200–8.

3. Böhm B, Palma M, Ousley J, Keane G. Competency-based mental health supervision: evidence-based tool needs for the humanitarian context – ERRATUM. Glob Ment Health (Camb). 9:249.

4. PS Centre Competency Framework for Psychosocial Support Delegates in Emergencies - Psychosocial Support IFRC. 2016. https://pscentre.org/?resource=competency-framework. Accessed 15 Sep 2023.

5. German Cooperation Deutsche Zusammenarbeit. Recommendation Paper on Training and Capacity Development in Mental Health and Psychosocial Support (MHPSS) in Development Cooperation As Exemplified in the Context of the Crises in Syria and Iraq. 2019.

6. IASC Guidelines on Mental Health and Psychosocial Support in Emergency Settings, 2007 | IASC. https://interagencystandingcommittee.org/iasc-task-force-mental-health-and-psychosocial-support-emergency-settings/iasc-guidelines-mental-health-and-psychosocial-support-emergency-settings-2007. Accessed 15 Sep 2023.


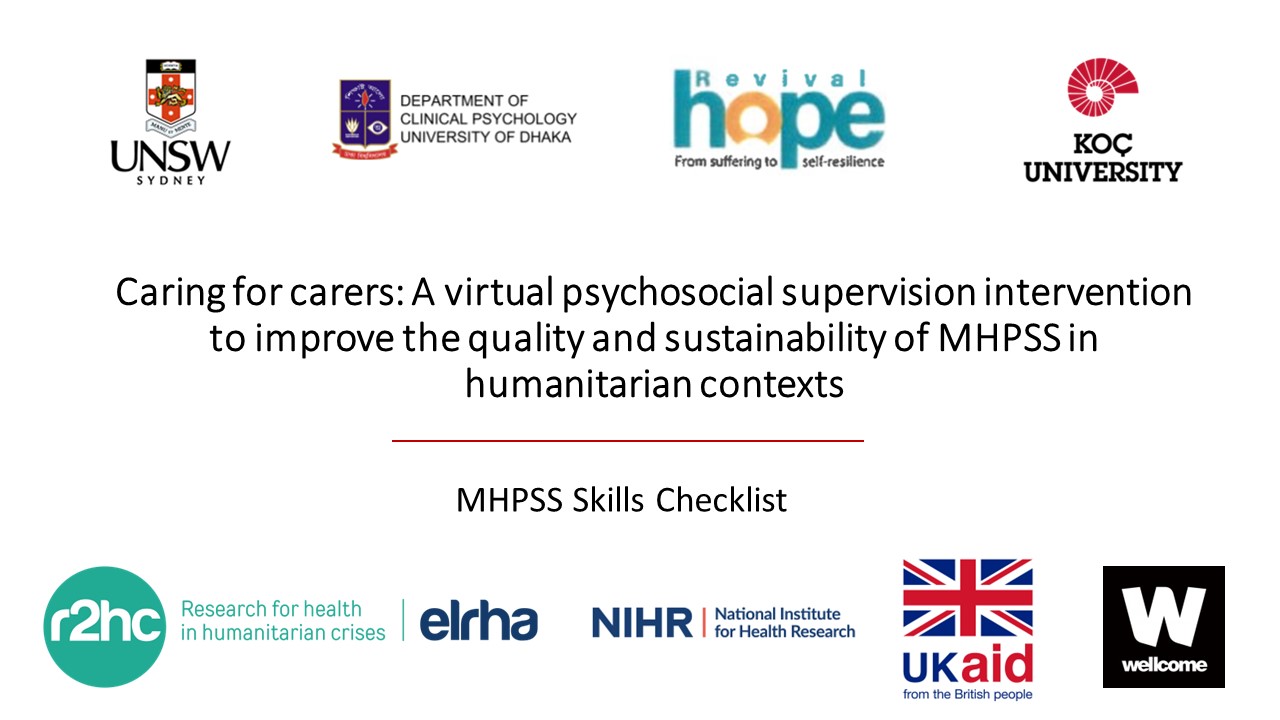

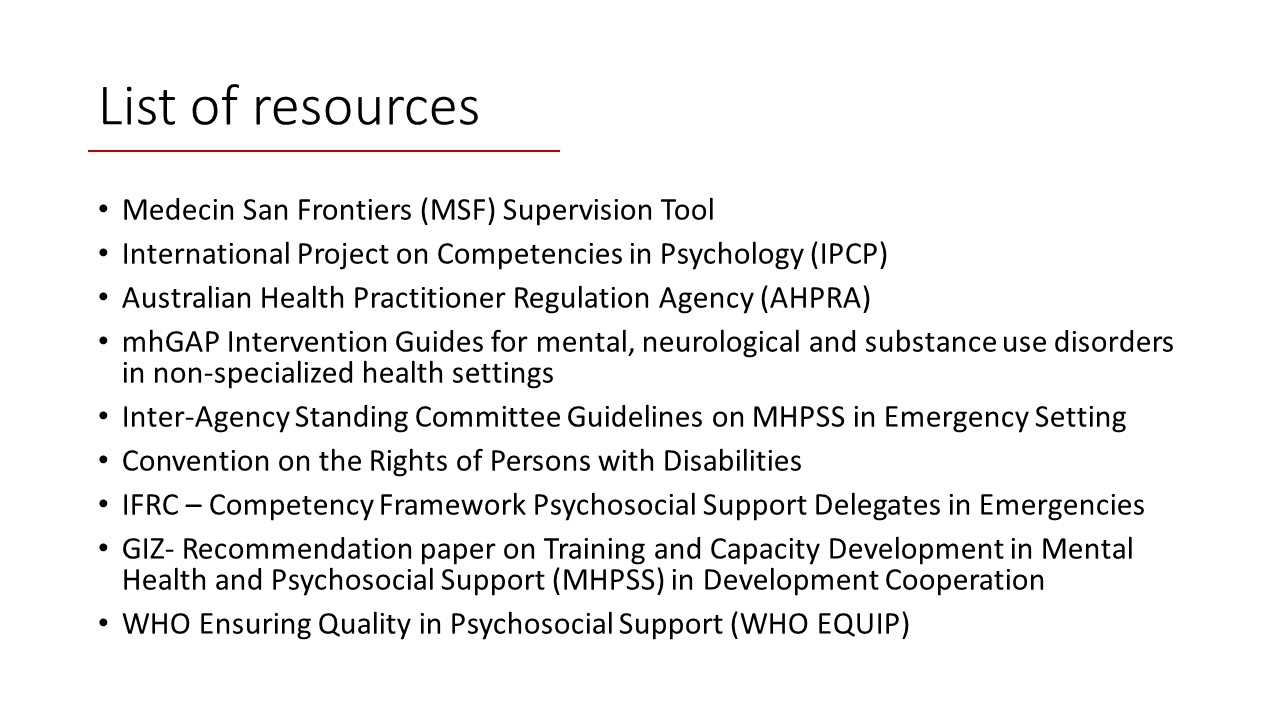


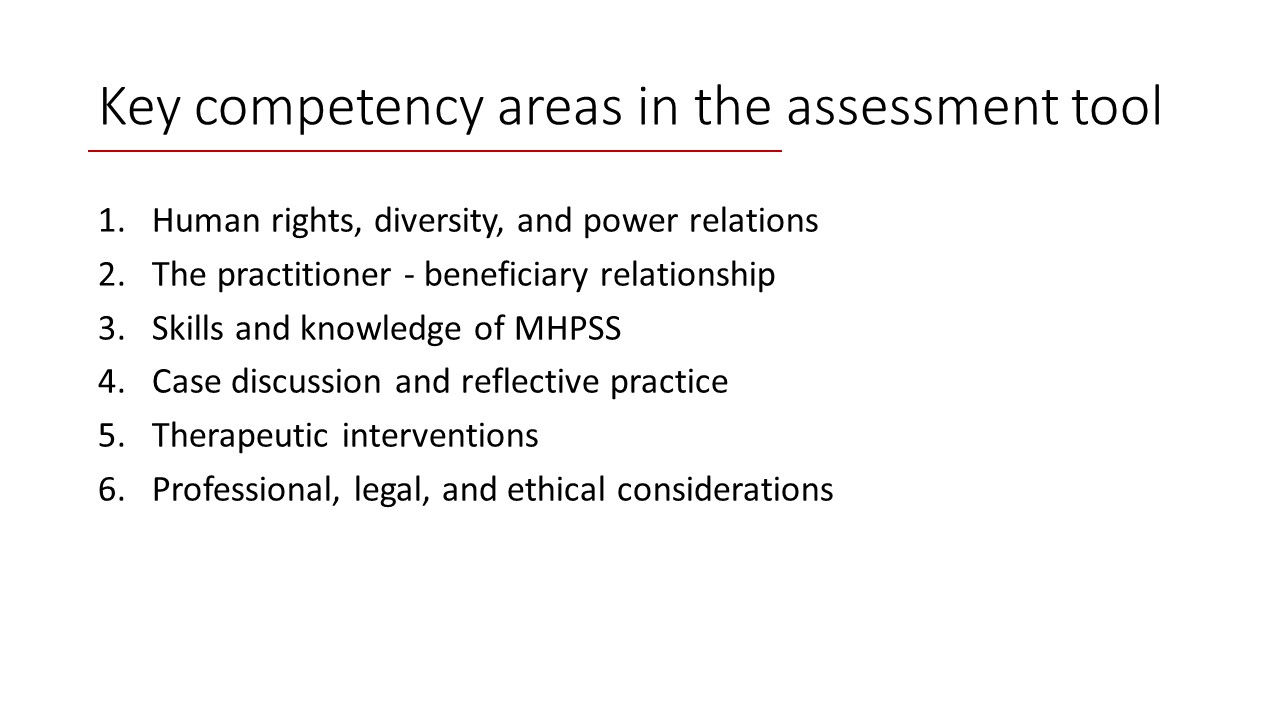

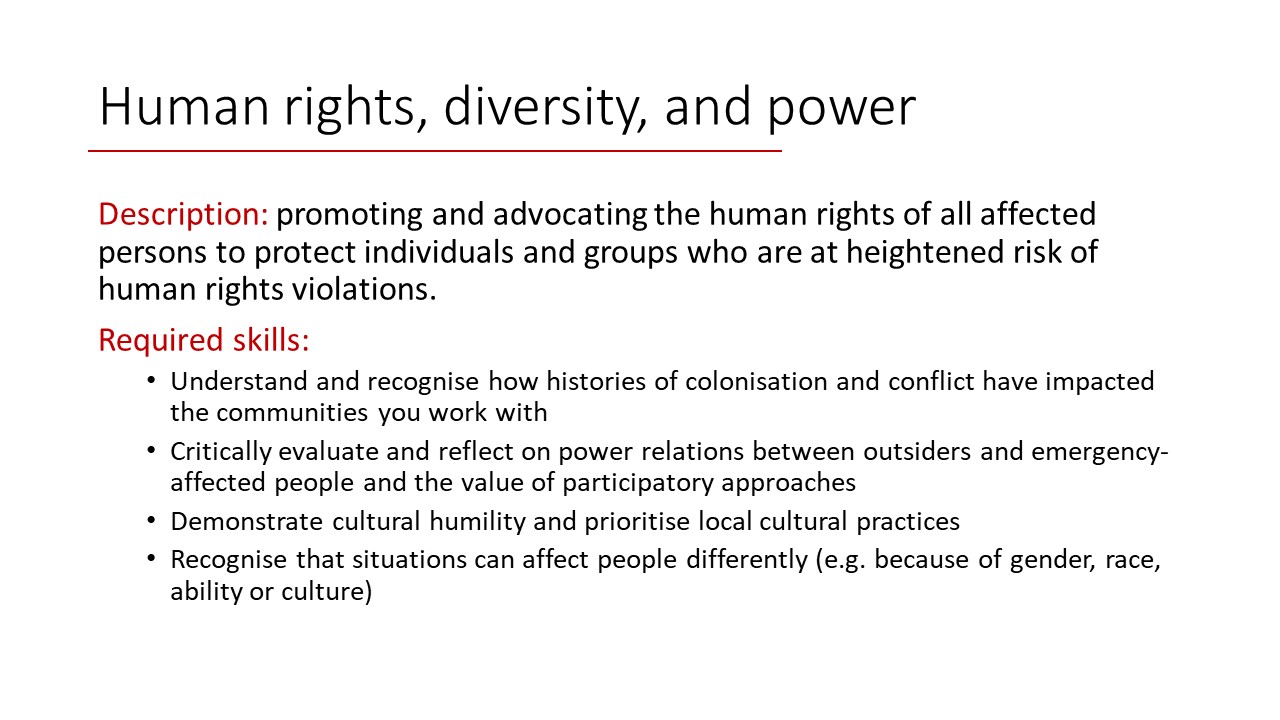

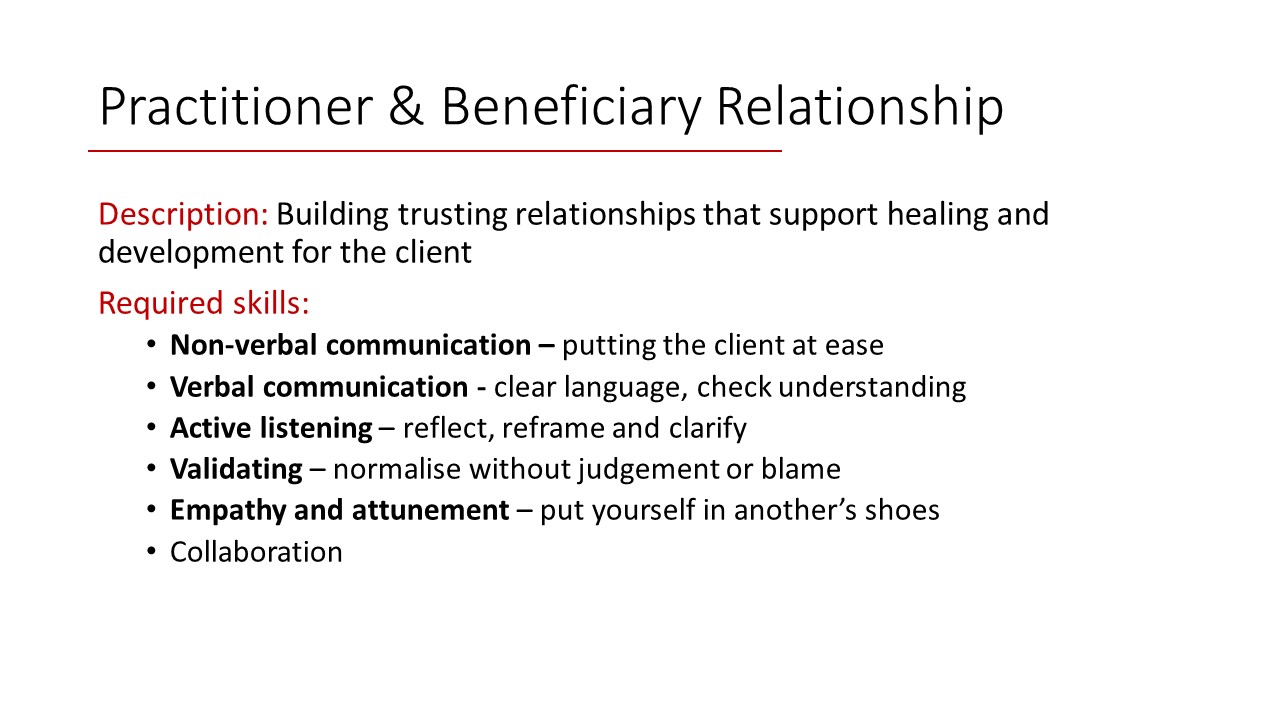

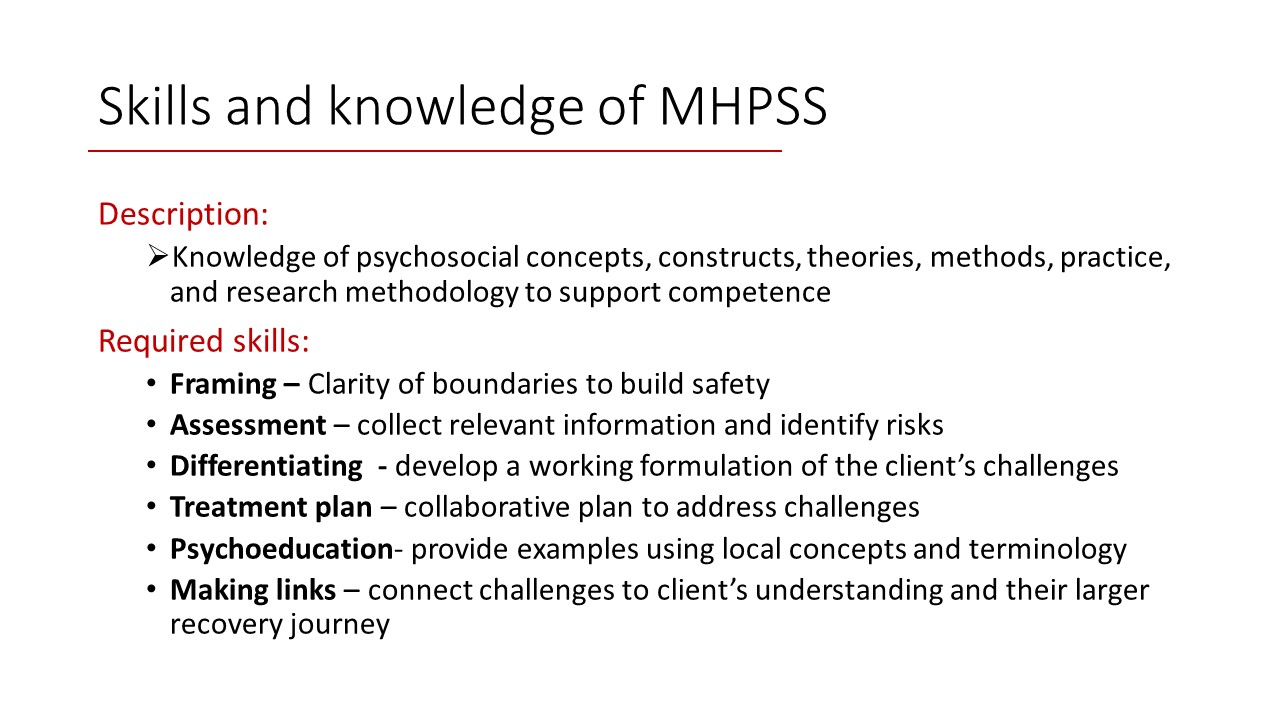

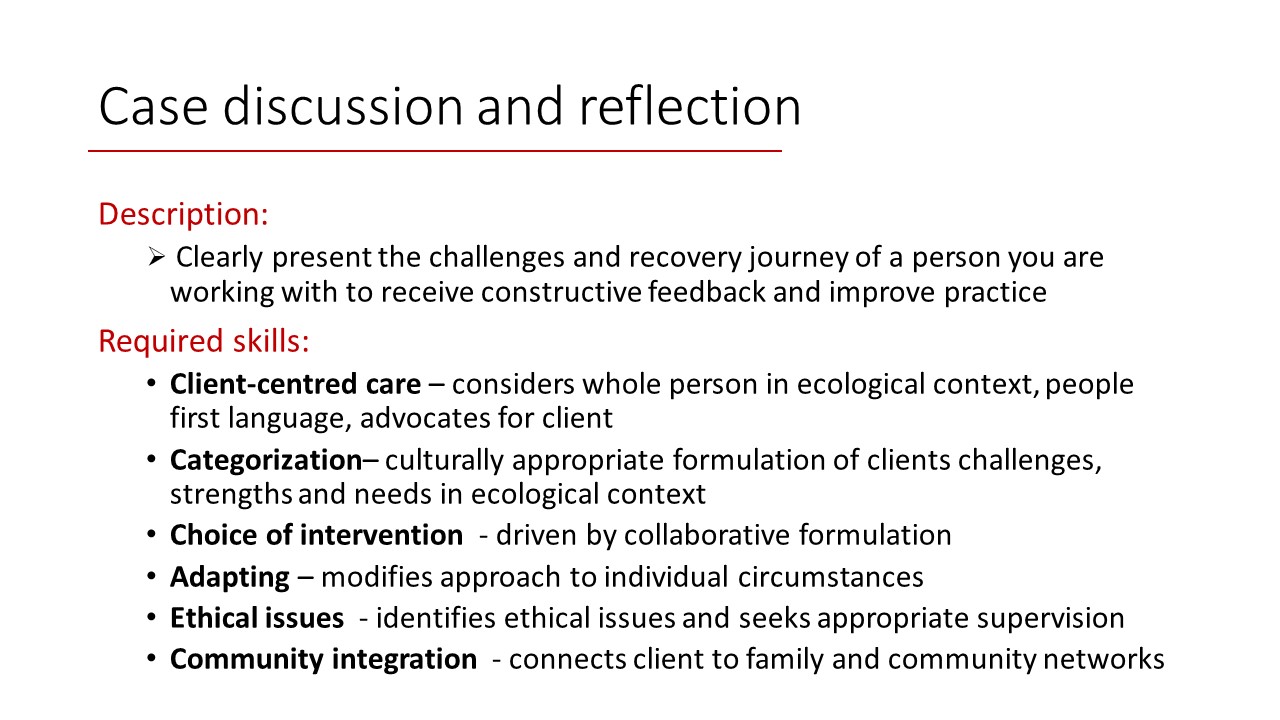

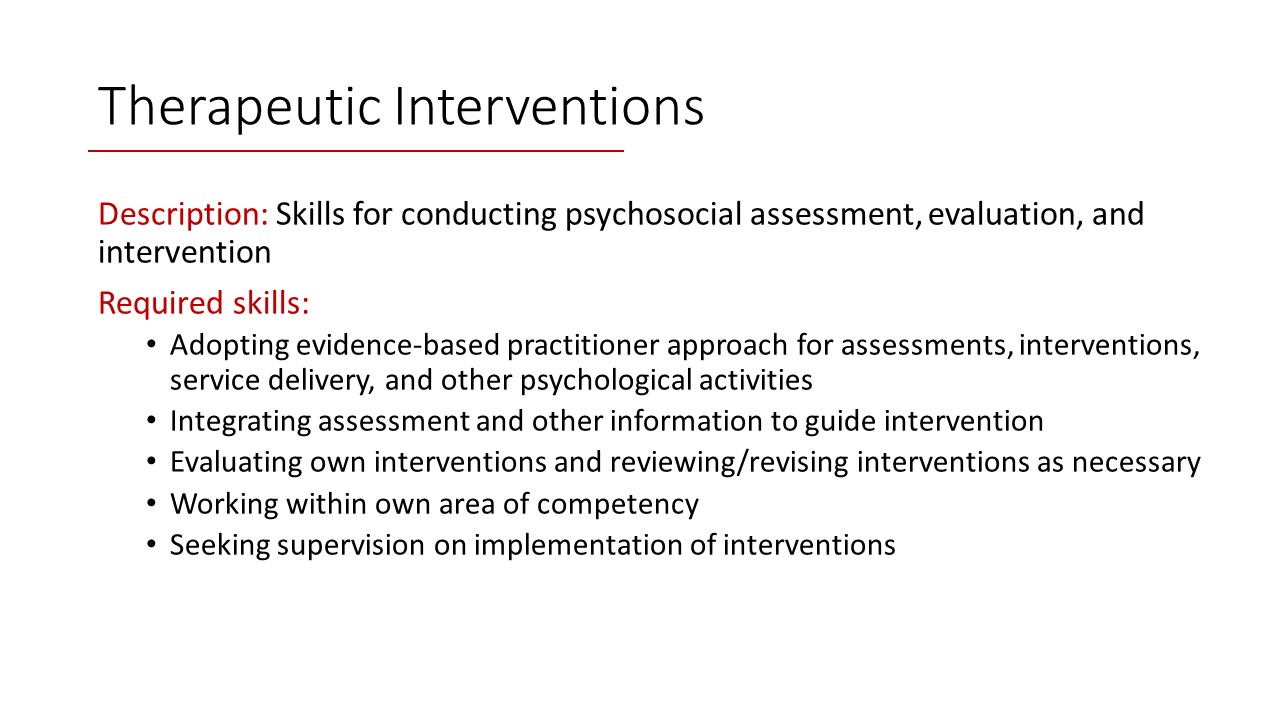

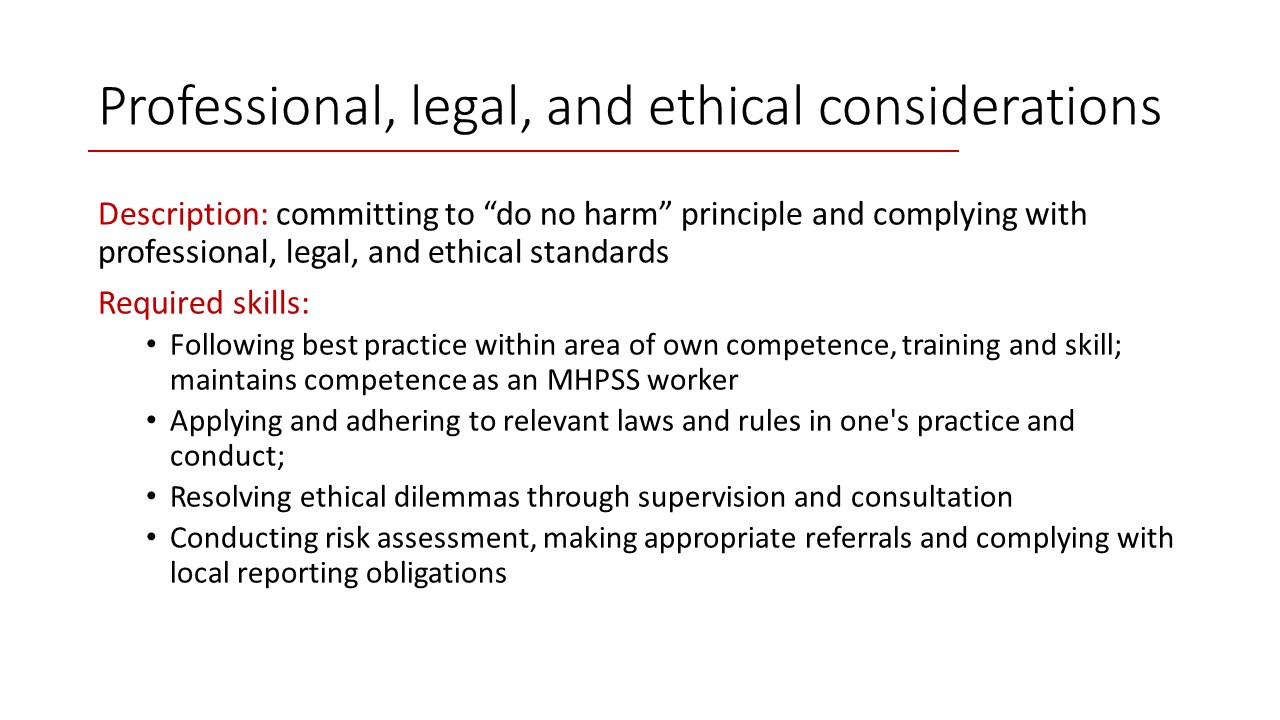


CARING FOR CARERS: A PSYCHOSOCIAL SUPERVISION INTERVENTION FOR MENTAL HEALTH PRACTITIONERS


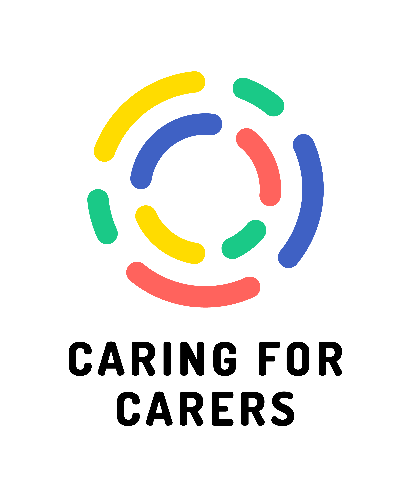


SUPERVISOR HANDBOOK

**Preface**

This handbook was prepared to provide guidance to the local and international supervisors to help them with group supervision facilitation. Although this book summarizes the key points and gives a list of useful documents for supervision, it does not aim to provide exhaustive information or guidance to the supervisors. We rely on the ever-changing relationship dynamics between and experiences of supervisors and supervisees in this exciting journey.

The following members of the Caring for Carers Project contributed to preparing the handbook. Please do not share with anyone outside the group until the supervision program is completed.

Scarlett Wong, [scarlett.wong@unsw.edu.au](mailto:scarlett.wong@unsw.edu.au)

Ruth Wells, [ruth.wells@unsw.edu.au](mailto:ruth.wells@unsw.edu.au)

Salah Lekkeh, [salah.lekkeh@hope-revival.ngo](mailto:salah.lekkeh@hope-revival.ngo)

Gülşah Kurt, [g.kurt@unsw.edu.au](mailto:g.kurt@unsw.edu.au)

Our partners:

University of New South Wales, Sydney Australia

Koc University, Istanbul, Türkiye

Hope Revival Organization, Gaziantep, Türkiye

Dhaka University, Dhaka, Bangladesh


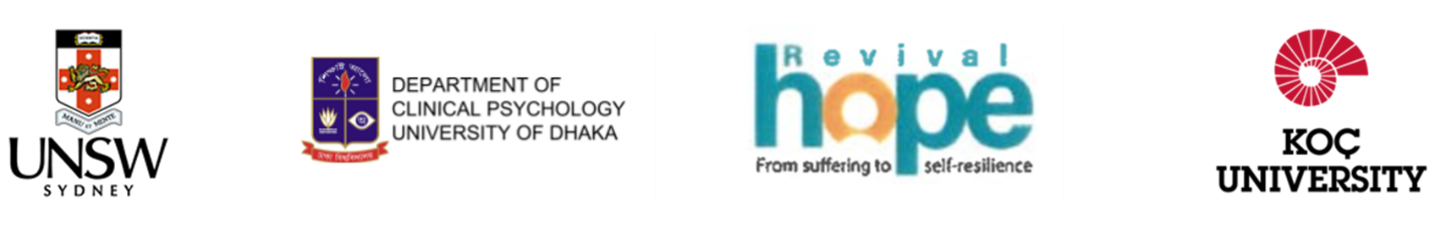


Our funding agencies:

This project is funded by Elrha’s Research for Health in Humanitarian Crises (R2HC) Program, which aims to improve health outcomes by strengthening the evidence base for public health interventions in humanitarian crisis. R2HC is funded by the UK foreign, Commonwealth and Development Office (FCDO), Wellcome, and the Department of Health and Social Care (DHSC) through the National Institute for Health Research (NIHR). The funding body had no role in the conceptualization; writing of this handbook; or the decision to submit the handbook for publication.


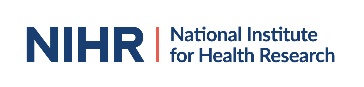

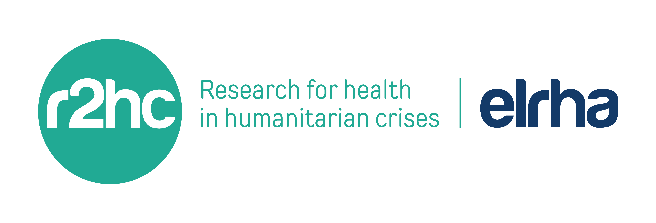

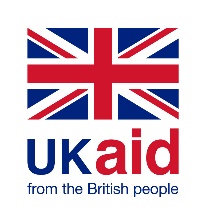

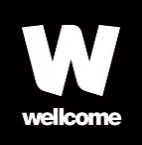


Contents

[1. POCKET SUMMARY 4](#_Toc115239985)

[1.1. Checklist 4](#_Toc115239986)

[1.2. Key Points 4](#_Toc115239987)

[2. GUIDED CO-SUPERVISOR CONVERSATION ON ZOOM 5](#_Toc115239988)

[2.1. Areas to cover: 5](#_Toc115239989)

[3. OVERVIEW OF PROGRAM STRUCTURE 6](#_Toc115239990)

[3.1. Pre-Supervision Program Resources 6](#_Toc115239991)

[3.2. UNSW Badge and Requirements 6](#_Toc115239992)

[3.3. CANVAS Platform 6](#_Toc115239993)

[3.4. Integrated Model for Supervision Handbook Chapters 7](#_Toc115239994)

[4. CONTEXT AND BACKGROUND 8](#_Toc115239995)

[4.1. Türkiye 8](#_Toc115239996)

[4.2. Northwest Syria 8](#_Toc115239997)

[5. INTRODUCTION TO GROUP SUPERVISION 10](#_Toc115239998)

[5.1. What constitutes ‘supervision’ in this program? 10](#_Toc115239999)

[5.2. First 1-3 Sessions 11](#_Toc115240000)

[5.4. Group Supervision Structure – Suggestions 12](#_Toc115240001)

[5.5. Co-Supervision Ideas 14](#_Toc115240002)

[6. SUPERVISION CONTRACT SAMPLES 15](#_Toc115240003)

[6.1. Sample 1 15](#_Toc115240004)

[2. Sample 2 19](#_Toc115240005)

[7. CASE PRESENTATION SAMPLES 21](#_Toc115240006)

[7.1. Sample 1 21](#_Toc115240007)

[7.2. Sample 2 22](#_Toc115240008)

[8. PRACTITIONER COMPETENCY TOOL 22](#_Toc115240009)

[9. SUPERVISION ALLIANCE STRATEGIES 23](#_Toc115240010)

[9.1. Supervision Alliance Scale 23](#_Toc115240011)

# 1. POCKET SUMMARY

## Checklist

- Have we done the Co-Supervisor Guided Conversation?
- Have we discussed the structure of the first 1-3 sessions?
- Have we agreed on Roles and Responsibilities as Co-Facilitators?
- Have agreed on the Templates we want to use?
- Have we discussed Group Rules and Group Contract?
- Have we agreed on feedback for each other and feedback from the group?
- Have we agreed on how to get supervision alliance feedback?
- Have we discussed Risk and Ethic-legal issues and how to manage this?

## Key Points

- **Co-Supervisor Relationship:**
  - Take time to get to know each other and discuss how you want to co-facilitate a safe and effective Group Supervision Space
- **Group forming and norming:**
  - Will take 1-3 sessions
- **Group Supervision Structure Suggestions:**
  - Box of Chocolates
  - IMS Handbook
- **Feedback Strategies:**
  - One word, One sentence, Supervisor Alliance Scale
- **Templates:**
  - Practitioner Competency Self-Assessment Tool,
  - Supervision Alliance Scale,
  - Case Presentation Template

# GUIDED CO-SUPERVISOR CONVERSATION ON ZOOM

**Introduction:** In the lead-up to the commencement of the Supervision Program, here are some conversation topics that might be helpful to discuss:

- Organise a time before the supervision session begin to meet with your translator to discuss the topics below
- Remember to Press Record on this Zoom Meeting!
- Please reflect on how many meetings you have had prior to this meeting and in what form (WhatsApp, Zoom, etc.)

## Areas to cover:

1. **Therapy Preference:**

- What kind of intervention, counselling model, technique, or strategies do we usually prefer in counselling?
- If our backgrounds and approaches are different, how will we manage our different approaches in supervision?

1. **Structure and Process:**

- How can we structure and/or share the roles in Group Supervision?
- If unsure, you can use the handbook for some ideas about co-facilitation and group supervision structures
- How will we know if there is an issue with supervision?
- How will we communicate with each other if we need to discuss an issue or challenge about the group?
- What are the needs of the translator, how do they want us to work together?

1. **Getting to know each other:**

- What are we curious about or would like to know about each other?
- What do we both think are the qualities of a good supervisor? And a not so good supervisor?

1. **Needs:**

- What information, help, or support do we both need, and how can support each other to achieve this?

1. **How do we each feel about supervising in this program?**

# OVERVIEW OF PROGRAM STRUCTURE

## Pre-Supervision Program Resources

MHPSS practitioners have been provided with resources on key areas of clinical practice such as human rights, counseling skills and relationships, and ethical issues. Written materials (e.g., journal articles and policy reports) were selected and then translated into Arabic and Bangla.

You can find all these materials on the CANVAS course site (see below for information about CANVAS). They are organized under the headings:

- - 1. Human rights, diversity, and power
    2. Practitioner–beneficiary relationship
    3. Skills and Knowledge of MHPSS
    4. Case Discussion and reflection
    5. Professional and Ethical issues

## UNSW Badge and Requirements

Practitioners participating in this program are offered access to UNSW Medicine Short Courses so that they can receive official accreditation of their learning. People who complete the short course will be able to get a micro-credential, which is like a university subject completed outside the structure of a degree. These courses can be counted towards a future degree – although access to a full degree is extremely unlikely for many of the people enrolled in this program because of their refugee status and the huge barriers that exist to migration for our colleagues. This micro-credential is a rare opportunity for them to receive official recognition from an international university. They will receive a digital badge which they can put on their LinkedIn on their CV.

## CANVAS Platform

You will receive an invitation to log into CANVAS (the online platform for the course), where you will find all the information that the students receive, as well as the links to the zoom meetings for your supervision sessions and online discussion boards for your group. Research assistants will share the link for navigating in CANVAS. This short video will guide you through the CANVAS platform and show how to use it as a course registrar.

You can log into CANVAS [here](https://unswmedicine.instructure.com/courses/149?invitation=uE0Qqgy3Zr1pi8prhQAk4dwb5306I2BzM6MgMPkK) and the course outline [here](https://unswmedicine.instructure.com/courses/149/pages/course-outline?module_item_id=2850).

The main points you need to know each practitioner is required to:

1. complete a case presentation during a supervision session
2. upload their completed case template at least three days before their presentation
3. submit a completed supervision and clinical practice log of their hours of supervision and supervised practice.

**Note:** Syrian co-supervisors will also be offered this micro-credential, which means they will also need to complete a case presentation.

## Integrated Model for Supervision Handbook Chapters

We are happy to share with you the Integrated Model for Supervision (<https://pscentre.org/?resource=integrated-model-for-supervision&selected=single-resource>) developed by the International Federation of Red Cross Red Crescent Societies. This is the first resource about supervision developed specifically for humanitarian settings and addresses a significant gap in the field.

This document is extremely helpful because it provides an introduction to supportive supervision for supervisors, organisations and supervisees. Most organisations and practitioners working in MHPSS in humanitarian settings have no or very limited experience with clinical supervision. Often the concept is confused with management supervision, so there is a lot of work to be done to help everyone understand the purpose of supportive supervision, which is focused purely on wellbeing and clinical development. It also provides a great framework for practitioners to help them understand what to expect in coming into supervision.

**We suggest using this framework as a jumping-off point for coming to a shared understanding with your group.**

**Chapter 3** provides a basic background for running supervision, including many concepts you are likely very familiar with.

**Chapter 4** is the section we have translated and shared with the supervisees. You can draw on this chapter to introduce topics such as case presentations, reflective practice and making use of feedback.

Of course- you will have your own style for supervision which we encourage you to use. We also hope that this framework provides some consistency across groups to help supervisees as they work through uncertainties in building trust in their new groups.

# 4.CONTEXT AND BACKGROUND

We have prepared a series of videos which aim to introduce you to the contextual information you will need to help you understand the context in which these clinicians work. We will continue to share resources with you to support development of cultural competence and help answer the questions which we are sure will come up for you.

We are also happy to meet with you if you need to discuss the complexities of working with people in these humanitarian crises – so please don’t hesitate to reach out if you have any questions.

## 4.1. Türkiye

Currently, Türkiye hosts the largest number of Syrians, with 3.7 million. More than 98 percent of Syrians live in the cities, mainly in Istanbul, Turkey^1^ (United Nations High Commissioner for Refugees, 2022). They are given a legal status called “temporary protection status,” which provides them access to basic services such as employment, education, and health. Despite this status, Syrian refugees are likely to experience a multitude of socio-economic (e.g., language barrier, discrimination, social isolation) and structural stressors (e.g., not being recognized as a refugee and difficulties with the asylum process) in daily life, all of which adversely impact their mental health^2,3^ (Acarturk et al., 2021; Kurt et al., 2022). Considering conflict and displacement-related stressors, mental health problems are highly prevalent among Syrians. Almost half are at risk of developing depression, anxiety, or posttraumatic stress disorder^3^ (Kurt et al., 2022).

## 4.2. Northwest Syria^4^

About 4.5 million are living in NWS, among them are 2.8 million IDPs. In other words, 20% of the Syrian population are living in an area that is less than 11% of the Syrian Arab Republic lands, in which MHPSS practitioners in NWS are striving to provide their services within this area with limited resources and capabilities, in line with the constant hostilities.

The majority of MHPSS interventions in NWS are sorted in the second and third levels (focused non-specialized) of the IASC Pyramid of MHPSS interventions, and the majority of MHPSS practitioners work as psychosocial support workers (PSW), which is a unique job title set by Syrian MHPSS specialists in the context of NWS, after developing a manual in 2017 dedicated to the rehabilitation, training, and supervision of these PSWs at the level of focused non-specialized interventions. The number of these workers at the beginning of 2022 was about 308 employees working in almost 15 organizations.

Given that the available psychiatrists and psychologists are very few in NWS, there are 2 specialized psychiatrists and 8 resident psychiatrists who have not completed their specializations yet, besides the availability of 25 to 50 clinical psychologists. The WHO developed the mhGAP Manual to scale mental health services in non-specialized health settings to achieve universal health coverage. In this context, almost 100 mhGAP practitioners provide psychotropics for the most common mental disorders listed in the mhGAP program, where WHO provides essential psycho-drugs to the organizations working in the MHPSS field in NWS according to the availability of funding sources or the active MHPSS projects.

Regarding clinical supervision, many initiatives have been launched, especially by the WHO, to provide supervision for both the mhGAP program, problem management plus (PM+), and the health and MHPSS workers after receiving suicide management training. Whereas the most important initiative to improve the supervision capacity, was launched by the German International Cooperation Agency (GIZ) through a training program lasted for two years from 2019 to 2020 to prepare 20 Syrian qualified supervisors, besides launching another training program in 2021 to qualify 20 new supervisors, and it is still ongoing.

The majority of MHPSS practitioners are working in primary health care centers, hospitals, community centers, or in mobile clinics that visit IDPs, while residents and specialized psychiatrists are working in specialized MHPSS centers or Hospitals. PSWs are providing group awareness sessions on MHPSS topics, mental disorders, and some group PSS programs for children and adults, in addition to conducting outreach visits to IDPs communities and homes to assess their cases, provide psychological first aid and psychological counselling, referral to the necessary required MHPSS services, where available, while following up on the cases that need psychotropics, in addition to providing counselling according to the PM+, which they were already trained on.

It is worth noting that most of the PSWs are graduated from the faculties of education, psychological counselling, or psychology, whereas the others are graduated from universities or other intermediate institutes. Taking into consideration that only graduates of psychology or psychological counselling are eligible to work as psychologists.

**Quotes from MHPSS practitioners:**

- *A psychological counselor in the countryside of Aleppo said, "Here, none of the specialists in psychological counseling and psychology has attended accredited training in a specific clinical field and completed a full supervision period!"*
- *“Being a PSW, is being a humanitarian worker in the first place. Consequently, my job is to raise awareness about issues related to MHPSS, such as diseases, disorders, and problems... I help people to access services... I help patients by applying the techniques I learned before to help them adapt positively. The most important characteristic of my work is my commitment to the principles of confidentiality, privacy, respect, discrimination, and equality.”*
- *My role is to provide support and assistance to people, help them to recover and enhance their ability to cope positively with difficult and new situations and provide group psychoeducation sessions to the community to raise their awareness about MHPSS issues.*

^1^United Nations High Commissioner for Refugees. (2022). UNHCR Turkey Bi-Annual Factsheet, <https://www.unhcr.org/tr/en/factsheets-and-dashboards>

^2^Acarturk, C., McGrath, M., Roberts, B., Ilkkursun, Z., Cuijpers, P., Sijbrandij, M., ... & Fuhr, D. C. (2021). Prevalence and predictors of common mental disorders among Syrian refugees in Istanbul, Turkey: a cross-sectional study. *Social psychiatry and psychiatric epidemiology*, *56*(3), 475-484.

^3^Kurt, G., Ventevogel, P., Ekhtiari, M., Ilkkursun, Z., Erşahin, M., Akbiyik, N., & Acarturk, C. (2022). Estimated prevalence rates and risk factors for common mental health problems among Syrian and Afghan refugees in Türkiye. *BJPsych Open*, *8*(5), e167.

^4^World Health Organization. (2021). NW Syria MHPSS Mapping October-December 2021. <https://app.powerbi.com/view?r=eyJrIjoiNTMwNjY0N2MtODE0Ni00N2JmLWEwYzEtYTM5ZjNmOGRjYTZjIiwidCI6ImY2MTBjMGI3LWJkMjQtNGIzOS04MTBiLTNkYzI4MGFmYjU5MCIsImMiOjh9>

# 5.INTRODUCTION TO GROUP SUPERVISION

## 5.1. What constitutes ‘supervision’ in this program?

Reflect on supervision you may have had.... it probably involved bringing up some issues you had with delivering and intervention or therapy technique, difficulties regarding your relationship with your patient/client/beneficiary, questions about why patients/clients/beneficiaries are not improving, ethical issues. Similar things will be brought up in this supervision program as well.

There will also be thing you may not be accustomed to. In some cultures, supervision is more didactic or about teaching. Also in some cultures, it is not as safe to show vulnerability, particularly in a group setting. It is also not always usual to be asked reflective questions.

Also, sometimes the supervisees will be psychosocial workers and not qualified psychologists or not allowed to work in the role of a psychologist. As such, it’s good to be clear with the supervisees about what they need from supervision and what is within their scope of responsibility.

Sometimes, supervision may involve sharing resources that you know about or demonstrating how you do certain types of techniques. It’s not uncommon for supervisees in other contexts with limited resources and access to training to ask for training on treating conditions, e.g., Trauma therapy. It’s ok to say upfront your experience and modalities that you feel confident working with. You can help and suggest information and send them links or articles you know about. In these contexts, they often do not have as much awareness due to the limited translated resources internationally.

**Your role as Supervisor can involve:**

- - Facilitating safety in the group
  - Reminding the group of respectful communication, rules, etc.
  - Prioritising the needs of the presenter during Case Presentation sessions
  - Directing “traffic.”
  - Being aware of members who may not have as much “airtime” and supporting them to participate if needed
  - Guiding discussions that are difficult
  - Giving reflections about the process when appropriate
  - Bringing your skills, expertise, and knowledge when there is a clear absence and need for your input
  - Using your wisdom and experience to support Group cohesion and safety
  - Keeping boundaries, like time management, topics that are able or not to be discussed, rules, etc

## 5.2. First 1-3 Sessions

In the lead up to beginning the supervision program, you will have hopefully met with your co-supervisor on-line a few times, had discussions on WhatsApp and gone through your guided conversation.

**Now you are ready to meet your group and begin group Supervision!**

The first few sessions are very important in establishing the norms, safety, structure and processes of the group. Given there are different cultures, languages, experience, exposure to group supervision, technical difficulties etc it will take time and patience to develop a relationship and understanding. In the first few sessions we suggest considering:

- Ice breaker and getting to know your activities
  - E.g., Go around and introduce self, one thing interesting about yourself, your favourite food
- Discussing and agreeing on the Group Supervision Contract (see Supervision Contract Samples for topics to consider)
- Discuss Goals
  - Individual and Group goals
- Group Rules
  - Safety
  - Confidentiality
  - Respect
  - Timeliness
  - Others?
- Discuss with the Content and structure of Group Supervision
  - E.g., cases for sessions 3-11, and then counselling skills for session 12-16
  - “Box of Chocolates” approach (see Group Supervision structure example)
- Go through a Case Template and Case Presentation example
  - Talk through and show the group how to present a case presentation
  - Encourage them to submit the case template to CANVAS at least 2 days prior to supervision in their chosen language so that you can put it into Google Translate if you need to
  - Reassure the group that they only need to fill out a few words for each section, and to spend no more than 15 minutes introducing the case so that all the time can be spent on receiving the support needed in the session
- Feedback and Alliance
  - Discuss ways that feedback can happen
  - E.g., You might give them 10 minutes at the end of the session to discuss:
    - 1 word to describe how you’re feeling
    - 1 sentence to describe anything new you’ve learned or thought about today
    - Fill out the anonymous Supervisor Alliance Scale for you as co-supervisors to discuss briefly after the session to improve or change future sessions
  - Email or WhatsApp for personal issues if necessary if you are ok with this
- Risk and Safety
  - In your Group Supervision contract conversations, you may consider discussing what to do if there is a risk or safety issue relating to themselves or a beneficiary. There is no one size fits all response, but in general, risk and ethic-legal issues should be resolved within the appropriate channels within the organisation the practitioner is a part of. However, Group Supervision can be used to discuss how to find the appropriate channels, role play or brainstorm ideas.

## 5.4. Group Supervision Structure – Suggestions

As you may know, there are pros and cons to Group Supervision compared to Individual Supervision. The pros include efficiencies of scale when there are limited resources, more ideas and sharing of resources when learning from others, support and normalisation of difficulties. The cons include less tailored individual time spent on individual difficulties and unsafe or unhelpful group dynamics.

A major benefit of group supervision relevant to cross-cultural contexts, especially when the supervisors are not so experienced with the clinical presentations in context, is that we can draw on the collective wisdom of the group t.

However, too often, if not well facilitated, the presenter can take the entire time just presenting the case, with no time to hear feedback or have their needs met for supervision. The idea of this structure below is to support the needs of each presenter each week. Ideally, one case is presented each week only.

**The suggested structure is the “box of chocolates” structure for case presentations:**

1. Supervisor A asks the Practitioner to briefly summarise the case (that they have already emailed or WhatsApped to the group) - 15 minutes
2. Supervisor A asks Presenter something like: “what are your needs for today” or “where are you stuck, needing more thinking space”....
3. Supervisor A asks each group member for clarifying questions, about the case to help them to form their answer. Presenter answers (15 minutes)
   1. Genuine questions not hidden directions, e.g, try to avoid asking, “why didn’t you try CBT”
4. Supervisor B is supporting with noticing questions, reminding about slowing down for translation, supporting requests by the group in the chat or the translator, noting the time, giving any reflections or support to Supervisor A, and noting down any suggestions for future learning/training, taking minutes if necessary
5. Supervisor B then asks everyone to think for a few minutes to write down some thoughts
6. Supervisor A then asks for a Round of Reflections:
   1. “let’s go around and each of us to offer a reflection or thought”
   2. “Remember, Presenter is asking for ___________ (remind of the stated need they said at the beginning)”
   3. Each member, including Supervisors, offer a reflection
      1. e.g. ‘I was wondering about whether this might be linked to the practical situation and their sense of security in their currently living situation, rather than your ability as a therapist’...
7. Supervisor B then suggests a few minutes for Presenter to think and reflect
8. Supervisor A then asks the Presenter, which offerings, or reflections would they like to comment on, or if any reflections or offerings helped to spark new thinking (like a box of chocolates, each reflection is uniqiue and helpful, and the presenter may want to choose one right now to pursue or that has triggered something for them to work with)
9. Presenter discusses any new thinking
10. If there is enough time, Supervisor B can let Supervisor A know that there is time for another round of reflections, based on what the presenter has said,
11. Supervisor A asks Presenter to ‘tie down any main reflections’.... asking them to reflect on any new directions, or main learnings new thinking from the session
12. Supervisor B then leads the last 10 minutes for Feedback (see Supervision Alliance Strategies section)

## 5.5. Co-Supervision Ideas

Co-supervision is a dynamic that not many practitioners or supervisors are accustomed to. Here are some considerations that you might like to discuss together on how to work together as co-supervisors.

1. You might want to consider whether you want to keep these roles throughout the program, whether you want to change it depending on different situations e.g. strengths, skills, knowledge experience etc
2. You might want to discuss how you will share and articulate the roles to the group. For example, you might want to say “Mary will be the timekeeper today, summarising responses, and taking feedback at the end. Salah will be asking the questions and facilitating the safety of the group through handling the questions from the group”.
3. You might want to consider how comfortable you both feel with different modalities and therapies, populations and presentations and other areas.
4. You might want to consider how to feel equally involved and equally respected in the program.
5. You might want to consider whether you’d like to meet up before or after supervision sessions, especially in the initial stages when you are working out a structure and norms.
6. You might want to agree on case presentation preparation, how far in advance you’d like cases sent to you and how translation will be done. E.g. the supervisee sends the template 1 day in advance in Arabic and this can be translated using Google translate by the Australian Supervisor.
7. You might want to consider how to create a thinking space within the session, or time for reflection, including time for you both to check in with one another on how you think the session is going and would like to alter it.
8. How do you want to handle different scenarios. E.g., if a group member is being overly critical of the presenter
9. Agree on how many and what sessions that you might want to have that are not related to case presentations e.g., a number of session on ACT, or psychodynamic
10. You might want to decide on who will take different roles and responsibilities:

- Who introduces the session and its structure (eg welcomes newcomers, answers the question, “What is the history of this meeting?”);
- Who facilitates a brief grounding exercise;
- Who directs ‘traffic’ and is the ‘lead’ in guiding the questioning
- Who is the timekeeper (usually Supervisor B – best seated opposite the clock in the room);
- Who facilitates the discussion toward the end of the supervision session about process.

# 6.SUPERVISION CONTRACT SAMPLES

## 6.1. Sample 1

**Supervision Agreement**

The  purpose  of  the  agreement/contract  is  to  ensure  safety  by  clarifying  goals,  boundaries,  role,  responsibilities,  assessment  and  reporting  procedures,  etc.  I  suggest  discussing  each  of  the  items  listed  below.

**Agreement  Between  Supervisor  And  Practitioner**

- **Purpose**  of  the  supervision  Identify  the  requirements  of  all  stakeholders  such  as  University,  Registration  board  and  Manager.  Also  elicit  the  practitioner's  needs  and  wishes  -­  there's  more  to  supervision  than  meeting  external  requirements!
- **Goals**  Short-­term,  specific  goals (SMART:  Specific,  Measurable,  Achievable,  Realistic,  Time  scheduled)  Break  down  the  requirements  and  wishes  into  manageable  chunks  and  specify  the  goals  on  a  timeline
- **Roles  and  Responsibilities** Who  is  responsible  for  what?  Be  clear  about  the  supervisor's  and  practitioner's  roles  and  responsibilities  in  each  of  the  supervisory  spaces  (Directive,  Evaluative,  Passive,  Restorative,  Active  and  Reflective;;  see  chapter  A3  in  *Reflective  Practice  in  Supervision*).  For  example,  the  practitioner  is  the  principle  explorer  and  the  supervisor  is  a  mindful  friend,  not  an  advisor,  when  in  Reflective  Space.
- **Accountability** Who  is  accountable  to  whom  for  what?  Be  clear  about  each  parties'  accountability  to  each  other  and  to  Manager  and  University/Board.
- **Assessment** What  will  be  assessed,  when  will  it  be  assessed  and  what  criteria  will  be  used**.** Due  process  requires  that  all  requirements  and  criteria  are  specified  clearly  from  the  start.
- **Reporting  processes**  to  Board,  Manager  and  University  What  reports?  To  whom?  When?  In  what  form?  Who  can  see  them?  What  are  the  limits  on  confidentiality?  What  are  the  mandatory  reporting  requirements?  A  statement  such  as  the  following  ensures  safety  within  legal/ethical  accountability:  *Everything  that  happens  in  supervision  is  completely  confidential  except  for  (1)  required  reports  to  University/Board/Manager  as  specified  in  this  agreement,  and  (2)  if  the  practitioner  practices  in  a  dangerous,  illegal  or  unethical  manner.*

*No  reports  will  be  made  without  giving  notice  to  the  practitioner.  The  practitioner  will  have  access  to  all  written  reports  before  submission  (with  right  of  response)  and/or  will  be  present  (physically  or  by  phone  conference)  during  verbal  reports.*

- **Methods**  What  methods  will  be  used  in  each  of  the  supervisory  spaces?  (e.g.  perhaps  live  observation,  roleplay  and  problem-­solving  will  be  used  in  Active  Space)
- **Therapeutic  orientation**  for  case  discussions  You  don't  have  to  be  rigid  about  the  approach  you're  using,  but  supervision  can  get  very  confusing  if  the  supervisor  is  using  e.g  a  psychodynamic  approach  and  the  practitioner  is  using  e.g.  a  CBT  approach.
- **Degree  of  self-­disclosure**  Different  therapeutic  orientations  and  different  supervisory  spaces  require  different  levels  of  self-­disclosure.  It  is  unethical  to  require  self-­disclosure  without  freely  given  informed  consent.  What  degree  of  self-­disclosure  is  needed  for  the  supervisory  approach  you  are  using?  Discuss  the  fine  line  between  reflective  supervision  and  therapy,  and  negotiate  that  either  of  you  can  voice  your  concerns  if  you  experience  that  the  process  is  crossing  the  line.
- **Record  keeping**  What  records  (such  as  log  books  and  session  notes)  are  required?  What  format?  Who  does  it?  How  will  they  be  safely  stored? Also,  what  information  will  go  into  client’s  files  regarding  supervision  discussions?
- **Policies**  to  be  followed.  What  legal  and  organisational  policies  must  be  followed?  What  about  procedures  for  when  external  requirements  haven't  been  set.  Examples  of  policies  to  be  identified  or  written:  how  to  respond  to  risk  of  harm  to  self  or  others,  intoxicated  clients,  weapons  or  threat  of  violence  during  sessions,  illegal  or  unsafe  behaviours  (such  as  non-­safe  sex),  home  visits,  use  of  touch,  working  alone  with  minors,  notification  of  suspected  abuse.
- **Schedule**  Times  and  place  of  supervision  and  who  books  formal  supervision  meetings  (especially  after  cancelled  sessions)
- **Emergency  back-­up**  Who  to  go  to  when  supervisor  is  unavailable?  (this  is  an  essential  item  for  supervision  of  trainees)
- **Informal  supervision**  arrangements.  Is  all  supervision  'formal'  or  is  it  also  available  'on  the  run'  (e.g.  as  you  pass  in  a  corridor)  or  during  a  social  tea  break?  I  put  'No  informal  supervision'  in  my  agreements.  The  practitioner  can  ask  for  a  quick,  formal  session,  but  I  don't  do  supervision  'on  the  run'.
- **Supervisory  alliance**  Ruptures  in  the  supervisory  alliance  can  undermine  supervision  and,  if  not  addressed,  have  the  potential  to  do  harm  to  the  practitioner.  On  the  other  hand,  ruptures  can  be  valuable  learning  opportunities  It's  best  to  discuss  the  potential  for  problems,  and  even  ruptures,  in  the  relationship  and  make  an  agreement  that  the  relationship  will  be  nurtured  and  any  concerns  will  be  addressed  as  quickly  and  safely  as  possible.  Also,  agree  on  what  to  do  if  either  party  has  concerns  that  can't  be  addressed  within  the  relationship.  Discuss  the  supervisor’s  role  as  a  ‘mindful  friend’  in  reflective  space,  and  clarify  the  boundaries  between  being  an  ally  and  forming  a  friendship.  Agree  on  regular  times  (perhaps  every  six  months)  to  review  the  supervisory  relationship  and  agree  that  either  party  can  ask  to  discuss  the  relationship  at  any  time.
- **Due  process** needs  to  be  followed  if  there  are  concerns  about  competence  or  professional  behaviour.  Specify  in  the  agreement  the  steps  that  will  be  taken.  For  example,  initial  alert,  then  highlight  issue,  then  strong  warning,  then  remediation  plan  with  support.
- **Reflective  practice** Acknowledge  that  reflective  supervision  can  be  unsettling  especially  when  it  queries  deep  assumptions  (including  the  assumptions  of  the  organisation).  How  will  painful  feelings,  such  as  shame,  and  discomfort  as  the  practitioner  approaches  their  'growing  edge',  be  shared  and  contained?  Negotiate  ways  the  supervisor  can  support  the  practitioner  through  the  process  if  they  choose  to  challenge  established  workplace  or  professional  cultures.
- **Fees**  The  'fee'  is  sometimes  money,  but  it  can  also  be  work  in  exchange,  or  the  satisfaction  of  nurturing  a  trainee's  professional  development,  or  challenging  one's  usual  practices,  or  learning  by  teaching.  Both  parties  need  to  be  clear  about  what  the  supervisor  needs  to  keep  them  enriched  and  engaged.
- **Review** dates  At  least  every  six  months.
- **Termination**  Date  when  this  supervision  agreement  will  terminate  or  when  a  new  agreement  will  be  negotiated

**Note:** Ideally, an agreement should also be negotiated with the management of the practitioner’s organization that addresses requirements, accountability, reporting, and limits on confidentiality.

**Sample Supervision  Group  Agreement**

This  agreement  is between  …………………………………………………..  (Supervisor) and (Supervisees) ……………………………………………………………………..

**Group Members’ Goals**

| Purpose | Consultation group  to  meet  Board's  CPD  requirements |
| --- | --- |
| Frequency | Fortnightly  -­  Feb  to  Nov  (about  22  meetings  in  the  year) |
| Duration | 2.5  hours |
| Times | 9  to  11.30am |
| Type of  group | Co-­operative/reflective  (i.e.  supervisor  will  facilitates  active  consultation  to  each  other  by  each  group  member) |
| Timetable | 9.00          Check  in/negotiate  agenda  9.15          Consultation  1  10.15      Consultation  2  11.15      Debrief  process/learning  statements/sign  logs  11.30      Close |
| Format | - Each  member  will  have  one  hour  in  alternate  meetings  to  focus  on  their  work  (about  10  hours  per  year) - If  a  member  has  an  urgent  issue,  they  can  negotiate  to  swap  time  with  another  group  member - Each  member  is  responsible  to  choose  their  content  and  tool  and  to  bring  5  copies  of  their  Notable  Incident  to  distribute |
| Venue | The  venue  will  rotate;;  each  group  member  will  host  every  fourth  meeting  and  provide  a  quiet,  private  space  as  a  meeting  room |
| Catering | The  host  will  provide  water/tea/coffee  throughout  the  meeting.  Each  member  brings  their  own  snacks. |
| Preparation | - Members  are  responsible  for  ensuring  they  are  meeting  Board  requirements  for  CPD  (plan  is  8  hrs  group  and  2  hrs  individual) - Members  will  acquire  skills  in  facilitating  reflective  practice - Members  will  come  to  meetings  prepared  for  their  hour |
| Confidentiality | - Except  in  the  event  that  dangerous,  illegal  or  unethical  behaviour  is  disclosed  (see  next  page)  everything  discussed  in  the  group  is  absolutely  confidential - Members  must  not  include  any  identifying  information  in  the  notes  they  take  during  the  meeting |
| Self-­  disclosure | •  Self-­disclosure  is  not  required.  Member  may  invite  relevant  personal  disclosure  from  others  but  each  person  has  the  right  to  refuse  to  discuss  personal  issues  in  the  group |
| Attendance | •  Each  group  member  will  attend  all  meetings  except  in  the  case  of  leave,  illness  or  emergencies |
| Responsibility and accountability  Assessment/reporting | - Each  group  member  is  responsible  for  their  own  work  and  is  accountable  only  to  themselves  and/or  their  manager;;  they  are  not  accountable  to  their  peers - The  group  members  are  not  responsible  for  the  work  of  other  group  members  and  do  not  have  the  right  to  give  instructions  or  to  follow-­up  on  outcomes - No  evaluation  or  reporting  will  occur  except  in  the  case  of  dangerous,  unethical  or  illegal  practice |
| Methods/Spaces | •  The  consultation  will  be  mainly  in  Reflective  Space,  using  the  principles  and  tools  of  reflective  supervision.  Where  needed,  problem  solving  in  Active  Space  and  debriefing  in  Restorative  space  will  be  provided.  It  is  not  expected  that  any  time  will  be  spent  in  Directive,  Evaluative,  or  Passive  Space |
| Safety, alliances and problems | •  All  group  members  appreciate  that  a  safe,  cohesive  group  is  needed  for  optimal  supervision.  Relationships  will  be  nurtured.  Members  will  deal  with  small  issues  before  they  escalate.  If  bigger  problems  arise,  the  group  members  will  vote  to  either:   - reduce  or  cancel  the  time  allocated  for  one  or  more                 consultations  in  a  regular  group  meeting  to  address                the  problem,  or   - schedule  an  extra  meeting  to  address  the  problem |
| Dangerous, Illegal  or  unethical  practice | - All  group  members  are  aware  of  the  mandatory  and  voluntary  notification  procedures.    Members  will  follow  due  process  in  that: - they  will  raise  any  concerns  they  have  about  another                 group  members  behaviour  as  soon  as  possible   - they  will  give  the  person  the  opportunity  to  remedy                 the  problem  (unless  it  is  a  mandatory  report  issue)            o  they  will  inform  the  person  that  that  are  intending  to                notify  before  the  notification  is  made   - All  group  members  appreciate  the  responsibility  of  other  members  to  abide  by  notification  standards  and  welcome  early  feedback  on  any  concerns  other  group  members  have  about  their  professional  functioning |
| Fees | •  The  supervisor  will  be  paid  $xx  per  session,  with  equal  contributions  by  each  of  the  four  group  members  (even  if  one  or  more  is  absent). |
| Duration | - This  contract  will  terminate  at  the  last  meeting  of  the  year - The  members  will  renegotiate  the  contract  at  the  first  meeting  next  year |

DATE:    ………………………………………    Supervisor  ………………………...……………….

Name…………………………………………    Signature  ……………………………………………

Name…………………………………………    Signature  ……………………………………………

Name…………………………………………    Signature  ……………………………………………

Name…………………………………………    Signature  ……………………………………………

## 2. Sample 2

This supervisory agreement is being agreed by……………(supervisee) and ……………(supervisor) and sets out the terms and objectives of the supervisory sessions that began on ……………. (date).

We have discussed the purpose of supervision together, including consideration of each of our expectations, and we have jointly agreed that the main purposes of supervision are as follows (please edit as necessary):

To provide………. (supervisee) with knowledge and information to improve their professional skills.

To collaboratively assess ………. (supervisee's) progress in developing his/her professional skills.

To provide support to ………. (supervisee) in all aspects of his/her work. The agreed frequency of meetings is: ………. The agreed duration of meetings is: ………. Describe how supervision will be delivered (e.g., remotely, in groups, individually): ……….

An agenda will be agreed upon by the supervisor and supervisee at the beginning of each session. A supervision record form will be used to document the main points of discussion in each session and any agreed actions. The final minutes of each session will be used to briefly discuss feedback on the session, such as what the supervisee has found useful in the session and what they would like more support with next time.

**Supervisor agreement**

I agree, to the best of my ability, to provide ………. with a safe and confidential space to discuss his/her work. I will provide information, guidance and support and will communicate feedback clearly and constructively. I will explain how progress will be measured. I will keep a record of each supervisory meeting that will be accessible to the supervisee.

**Supervisee agreement**

I agree that I will reflect on my practice before supervision sessions, and bring any issues arising in my practice to supervision at the earliest possible opportunity. I will do my best to integrate the feedback provided within supervision sessions into my practice, and I will ask questions when anything is unclear. If either the supervisor or supervisee have concerns that cannot be resolved within supervision, the course of action is: ……….

In the event of an emergency, the supervisee agrees to contact supervisor. If not available, then contact ………. (alternative contact name).

This agreement may be revised as needed, upon the request of either the supervisee or the supervisor, but only with the consent of the supervisee and approval of supervisor.

**Declaration of supervisor and supervisee**

We agree, to the best of our ability, to uphold the guidelines outlined in this supervision agreement.

………. ……….

Supervisor Supervisee

As a representative of the organisational management, I guarantee that I will endeavor to protect the time and space to enable supervision to take place.

………. ……….

Organizational representation Date

# 7.CASE PRESENTATION SAMPLES

## 7.1. Sample 1

| **Supervision Session Form**  **Case Formulation** |
| --- |
| Name of the presenter:  Date: |
| Client Code: |
| How many sessions have you seen the client? |
| When did you last see the client? (date) |
| Why are you presenting the case (e.g., where do you feel stuck?) |
| What input would you like today? (e.g., to have someone listen to me only, to give advice, to help think of ideas) |
| Presenting Problem (why did the client come to seek your help, what issues do you think they need help with)? |
| Who is in the family that is involved or important to this case? e.g., Mother, Father, children, brothers, sisters, grandparents.... |
| Your understanding of the presenting problems (how do you understand the problem the client is having, what do you think is affecting the problem e.g., grief and loss, lack of support, avoidance) |
| Hypothesis/Formulation (what are your hypotheses about the presenting problem, diagnoses, counseling planning, and goals) |
| Progress of counseling (how have you worked with the client to date, what have been the main issues addressed, models of counseling you have used, other issues |
| Reflections on counselor functioning (e.g., how are you feeling about your sessions, what do you think you did well, what do you think you would like to improve, what did you feel uncomfortable about, what did you feel happy about?) |

## 7.2. Sample 2

| **Supervision Session Form**  **Case Formulation** |
| --- |
| **Name of the presenter:**  **Date:** |
| **Goals for supervision** (why you are presenting this case, where you are stuck, what input you would like, what clinical issues you brought up here for supervision)**:** |
| **Reason for referral, referral info, specific contextual issues:** |
| **Current hypothesis / formulation/ case conceptualization:** |
| **Context: Any outside events affecting practitioner functioning or sessions** (e.g. fire, funding related issue, monsoon, flood or other disruption) |
| **Reflections on practitioner functioning:** |
| **Genogram/ background and family-related information of the case:** |

# 8. PRACTITIONER COMPETENCY TOOL

We have developed a practitioner competency tool to support practitioners in identifying which areas of their practice they would like to focus on developing. The tool is explicitly designed for their eyes only (unless they choose to share it with someone else) and is not an assessment or exam. We hope that tool provides a common framework for discussing competencies. In particular, we hope that it encourages practitioners to think beyond gaining knowledge of specific modalities (e.g., CBT, EMDR) to developing reflective practice about their own counselling skills, ethical considerations, human rights practice and relationship building. You will find the tool on the CANVAS website.

# 9. SUPERVISION ALLIANCE STRATEGIES

In order to gain feedback and/or get a sense of how the sessions are going, for you to be able to adjust your session, here are some suggestions:

Leave 10 minutes at the end of the session for everyone to:

- 1. Say one thing that you will take away from the session
  2. In a couple of words, say how you feel about today
  3. Complete the supervisor alliance scale on Zoom Poll (either anonymously or not) and ask for them submit to zoom poll. Then you both can meet after the session for 15 minutes to discuss the feedback and decide if you need to change process for the following session, bring it up in the group for discussion or some other resolution

You can model this by starting with yourselves e.g., “one thing that I’m taking away today is even though it is complicated to treat xyz, there are creative ways to get help... and a word that describes how I feel about today is ‘curious’ ”

## 9.1. Supervision Alliance Scale

After the session: please rate where today’s session was for you on the below-given poles.

**High challenge Low challenge**

**High support Low support**

|  | **Approach** |  |
| --- | --- | --- |
| This supervision session was not focused. |  | This supervision session was focused. |
|  | **Relationship** |  |
| My supervisor and I did not understand each other in this session. |  | My supervisor and I understood each other in this session. |
|  | **Meeting my needs** |  |
| This supervision session was not helpful for me. |  | This supervision session was helpful for me. |
