# Supplementary material for: Testing the effectiveness and acceptability of online supportive supervision for mental health practitioners in humanitarian settings: a study protocol for the caring for carers project
Source: BMC Psychiatry. 2023 Nov 28;23:884. doi: 10.1186/s12888-023-05246-1 (PMC10683137; doi:10.1186/s12888-023-05246-1)
Supplement: Supplementary file 2 — Supplementary Material 2: C4C Project R Workbook Setup. [file 12888_2023_5246_MOESM2_ESM.pdf]

Supplementary File 2

Code

Caring For Carers (C4C) : Protocol Paper

Louis Klein

Updated: 17 May, 2023[1]

Workbook setup

Hide

```
louisdesu::using("tidyverse","louisdesu","ids","stringdist","quanteda","c4c")
```

```
[1] "Loading: tidyverse, louisdesu, ids, stringdist, quanteda, c4c"
```

Hide

```
set.seed(2132) # a different seed was used for the actual project
```

Syria/Türkiye

example availability matrix

- this shows the data format prior to deidentification, which encodes sample work schedules for participants recruited into the C4C program
- note that work availability patterns vary both within participant and across participants for e.g., depending on which days are culturally treated as weekends

Hide

```
temp_matrix <- read_csv(file.choose(),show_col_types=FALSE)
temp_matrix[1:2] <- "[redacted]"
temp_matrix
```

organisation_name <chr>	practitioner_name <chr>	sunday <dbl>	monday <dbl>	tuesday <dbl>	wednesday <dbl>	thursday <dbl>	friday <dbl>	saturday <dbl>
[redacted]	[redacted]	0	1	0	1	0	0	0
[redacted]	[redacted]	1	1	1	1	1	0	1
[redacted]	[redacted]	1	1	1	1	1	0	1
[redacted]	[redacted]	1	1	1	1	1	0	1
[redacted]	[redacted]	1	1	1	1	1	0	1
[redacted]	[redacted]	0	1	1	1	1	0	0
[redacted]	[redacted]	0	0	1	1	1	0	0
[redacted]	[redacted]	0	1	1	1	0	0	0
[redacted]	[redacted]	1	1	1	1	1	0	1
[redacted]	[redacted]	1	1	1	1	1	0	1

1-10 of 33 rows Previous 1 2 3 4 Next

blindly assign practitioner and organisation IDs using `ranid()` function

- note that separate calls to `ranid()` are made to generate deidentified ID strings with different properties

Hide

```
(syria <- read_csv(file.choose(),show_col_types=FALSE) %>%
  rowid_to_column() %>%
  # blindly assign ranids to practitioners
  full_join(by="rowid",
    ranid(target_n = 33,
      dist_method = "lv",
      str_length = 12)) %>%
  rename("practitioner_id"=ranids) %>%
  group_by(organisation_name) %>%
  mutate(rowid = cur_group_id()) %>%
  # blindly assign ranids to organisations
  full_join(by="rowid",
    ranid(target_n = 8,
      max_symbols = 4,
      dist_method = "lv",
      str_length = 18)) %>%
  rename("organisation_id"=ranids) %>%
  ungroup() %->% tmp %>%
  select(organisation_id,practitioner_id,sunday:saturday))
```

organisation_id <chr>	practitioner_id <chr>	sunday <dbl>	monday <dbl>	tuesday <dbl>	wednesday <dbl>	thursday <dbl>	friday <dbl>	saturday <dbl>
31615cf4ab8cf46473	e800013c0847	0	1	0	1	0	0	0
0c32d0b43bbe0eff84	4aa2133f826c	1	1	1	1	1	0	1
0c32d0b43bbe0eff84	47a3a3095dec	1	1	1	1	1	0	1
0c32d0b43bbe0eff84	f117d25c0a66	1	1	1	1	1	0	1
0c32d0b43bbe0eff84	208d629694a7	1	1	1	1	1	0	1
0c32d0b43bbe0eff84	8e7dbe139a24	0	1	1	1	1	0	0
0c32d0b43bbe0eff84	1e6dfd6976bb	0	0	1	1	1	0	0
0c32d0b43bbe0eff84	ebd07064549b	0	1	1	1	0	0	0
0c32d0b43bbe0eff84	0708281ef145	1	1	1	1	1	0	1
0c32d0b43bbe0eff84	9431f62ac665	1	1	1	1	1	0	1
9a7c56a556@4402df7	fb037fdea0d3	1	1	1	1	1	0	0
9a7c56a556@4402df7	4e05cc076587	1	1	1	1	1	0	0
9a7c56a556@4402df7	bfdfcab06575	1	1	1	1	1	0	0
9a7c56a556@4402df7	81bcff38a98f	1	1	1	1	1	0	0
9a7c56a556@4402df7	fba0505e670d	1	1	1	1	1	0	0
87f00d883feba32%c6	0c002e7f7922	0	1	1	1	0	0	1
fc2dfc9e1eb8eded1e	71185e5f7c00	0	1	1	1	1	1	0
fc2dfc9e1eb8eded1e	e82bce28f935	1	1	1	1	1	0	1
fc2dfc9e1eb8eded1e	77a0f7030125	1	1	1	1	1	0	1
fc2dfc9e1eb8eded1e	d5ae0e9d2a8d	1	1	1	1	1	0	1
e0c14a5db8e43ed6e3	6585b5b24d6a	0	1	1	1	0	0	0
e0c14a5db8e43ed6e3	802911cec9a5	1	1	1	1	1	0	1
e0c14a5db8e43ed6e3	01cb69d07440	1	1	1	1	1	0	0
a8a5023d4e3f1b58@7	bd2a6d0b8f36	1	1	1	1	1	0	0
a8a5023d4e3f1b58@7	957f7a210030	1	1	1	1	1	0	0
a8a5023d4e3f1b58@7	6ab83860383b	1	1	1	1	1	0	0
2a624f486378fb325b	5b07276b8858	0	1	1	1	1	0	0
2a624f486378fb325b	c75a0456a83b	0	1	1	1	1	1	0
2a624f486378fb325b	a72468df5cfb	0	1	1	1	1	0	0
2a624f486378fb325b	5987bd443878	1	1	1	1	1	0	1
2a624f486378fb325b	77440812a765	1	1	1	1	1	0	1
2a624f486378fb325b	9c809dbe8d98	1	1	1	1	1	0	1
2a624f486378fb325b	3bcf78369d45	1	1	1	1	1	0	0

1-33 of 33 rows

Hide

```
# write_csv(tmp,"temp.csv")
rm(tmp)
```

- by writing the deidentified output to .csv and then calling `rm()` in the same step, the statistician remains blinded throughout the deidentification process

make days-per-week availability matrix

- in order to sample days using the `counterbalance_across()` function from the `c4c` library, availabilities from the deidentified matrix are recoded into character vectors with human-readable names

Hide

```
(syria <- syria %>%
  group_by(practitioner_id) %>%
  mutate(sunday = case_when(sunday==1~"sunday",TRUE~NA_character_),
         monday = case_when(monday==1~"monday",TRUE~NA_character_),
         tuesday = case_when(tuesday==1~"tuesday",TRUE~NA_character_),
         wednesday = case_when(wednesday==1~"wednesday",TRUE~NA_character_),
         thursday = case_when(thursday==1~"thursday",TRUE~NA_character_),
         friday = case_when(friday==1~"friday",TRUE~NA_character_),
         saturday = case_when(saturday==1~"saturday",TRUE~NA_character_)) %>%
  unite("availability",sunday:saturday,na.rm=TRUE,remove=TRUE,sep=",") %>%
  mutate(availability = str_split(availability,pattern=",")))
```

organisation_id <chr>	practitioner_id <chr>	availability <list>
31615cf4ab8cf46473	e800013c0847	<chr [2]>
0c32d0b43bbe0eff84	4aa2133f826c	<chr [6]>
0c32d0b43bbe0eff84	47a3a3095dec	<chr [6]>
0c32d0b43bbe0eff84	f117d25c0a66	<chr [6]>
0c32d0b43bbe0eff84	208d629694a7	<chr [6]>
0c32d0b43bbe0eff84	8e7dbe139a24	<chr [4]>
0c32d0b43bbe0eff84	1e6dfd6976bb	<chr [3]>
0c32d0b43bbe0eff84	ebd07064549b	<chr [3]>
0c32d0b43bbe0eff84	0708281ef145	<chr [6]>
0c32d0b43bbe0eff84	9431f62ac665	<chr [6]>

1-10 of 33 rows Previous 1 2 3 4 Next

apply `counterbalance_across()` to days-per-week availability matrix

- availability character vectors are then randomly sampled within participants but counterbalanced across participants

Hide

```
(syria_assignment <- syria %>%
  regroup(practitioner_id,organisation_id,availability) %>%
  counterbalance_across(list_name = availability,print_runs = FALSE) %>%
  mutate(morning = 0,evening = 1) %>%
  unite("times",morning:evening,na.rm=TRUE,remove=TRUE,sep=",") %>%
  mutate(times = str_split(times,pattern=",")))
```

practitioner_id <chr>	organisation_id <chr>	availability <chr>	times <list>
e800013c0847	31615cf4ab8cf46473	wednesday	<chr [2]>
4aa2133f826c	0c32d0b43bbe0eff84	thursday	<chr [2]>
47a3a3095dec	0c32d0b43bbe0eff84	thursday	<chr [2]>
f117d25c0a66	0c32d0b43bbe0eff84	tuesday	<chr [2]>
208d629694a7	0c32d0b43bbe0eff84	monday	<chr [2]>
8e7dbe139a24	0c32d0b43bbe0eff84	monday	<chr [2]>
1e6dfd6976bb	0c32d0b43bbe0eff84	tuesday	<chr [2]>
ebd07064549b	0c32d0b43bbe0eff84	tuesday	<chr [2]>
0708281ef145	0c32d0b43bbe0eff84	thursday	<chr [2]>
9431f62ac665	0c32d0b43bbe0eff84	wednesday	<chr [2]>

1-10 of 33 rows Previous 1 2 3 4 Next

apply `counterbalance_across()` to times-per-day availability matrix

Hide

```
syria_assignment %>%
  counterbalance_across(list_name = times,
                        print_runs = FALSE) %>%
  mutate(times = case_when(times==0~"morning",times==1~"evening")) %->% tmp
```

practitioner_id <chr>	organisation_id <chr>	availability <chr>	times <chr>
e800013c0847	31615cf4ab8cf46473	wednesday	morning
4aa2133f826c	0c32d0b43bbe0eff84	thursday	evening
47a3a3095dec	0c32d0b43bbe0eff84	thursday	morning
f117d25c0a66	0c32d0b43bbe0eff84	tuesday	morning
208d629694a7	0c32d0b43bbe0eff84	monday	evening
8e7dbe139a24	0c32d0b43bbe0eff84	monday	morning
1e6dfd6976bb	0c32d0b43bbe0eff84	tuesday	morning
ebd07064549b	0c32d0b43bbe0eff84	tuesday	evening
0708281ef145	0c32d0b43bbe0eff84	thursday	morning
9431f62ac665	0c32d0b43bbe0eff84	wednesday	evening

1-10 of 33 rows Previous 1 2 3 4 Next

Hide

```
  # %>% write_csv("BeneficiaryAssignment_Sun17-Sat23-Apr2021.csv")
```

assign practitioners recruited after `B-p1` timepoint and batch beneficiary interview assignment sheets

- as recruitment occurred throughout the Active Control period, newly recruited practitioners had to be included in successive assignments

Hide

```
library(doParallel)
registerDoParallel(cores=4)

for (i in seq_along(7:10)) {
# manually add newly recruited participant(s)
syria_temp <- ungroup(syria) %>%
  add_row(organisation_id = "c2f31!adeca8fa!707",
          practitioner_id = "89aaec256280",
          availability = list(c("sunday","monday","tuesday"))) %>%
  add_row(organisation_id = "6!91852b16514a4162",
          practitioner_id = "0c48f7945051",
          availability = list(c("sunday","monday","tuesday","wednesday","thursday","friday","saturday"))) %>%
  add_row(organisation_id = "ca94!88f0c677b7*29",
          practitioner_id = "707d234e5149",
          availability = list(c("sunday","monday","tuesday","wednesday","thursday"))) %>%
  add_row(organisation_id = "ca94!88f0c677b7*29",
          practitioner_id = "6a15c1ac4fee",
          availability = list(c("sunday","monday","tuesday","wednesday","thursday"))) %>%
  add_row(organisation_id = "384fd7da40@30ea6e8",
          practitioner_id = "a9d760bb5115",
          availability = list(c("wednesday","thursday"))) %>%
  add_row(organisation_id = "5ef914bde2d1fc@75a",
          practitioner_id = "31f4ba8b5968",
          availability = list(c("monday","tuesday","wednesday","thursday","friday")))

syria_temp %<>%
  # day assignment
  regroup(practitioner_id,organisation_id) %>%
  counterbalance_across(list_name = availability,print_runs = TRUE,threshold=1.5) %>%
  # assign default times to all practitioners
  mutate(morning = 0,evening = 1) %>%
  unite("times",morning:evening,na.rm=TRUE,remove=TRUE,sep=",") %>%
  mutate(times = str_split(times,pattern=",")) %>%
  # time assignment
  regroup(practitioner_id,organisation_id,availability) %>%
  counterbalance_across(list_name = times,print_runs = TRUE,threshold=1.5) %>%
  mutate(times = case_when(times==0~"morning",times==1~"evening"))

syria_temp %>%
  rowid_to_column() %->% tmp
  # write_csv(paste0("BeneficiaryAssignment_",i,".csv"))

tmp
}
```

```
[1] 4.593474
[1] 3.72827
[1] 2.073644
[1] 2.225395
[1] 3.309438
[1] 5.191568
[1] 3.505098
[1] 3.598942
[1] 3.146427
[1] 2.878492
[1] 3.146427
[1] 3.271085
[1] 4.969909
[1] 2.636737
[1] 2.588436
[1] 4.037326
[1] 2.664583
[1] 3.207135
[1] 3.735289
[1] 2.992053
[1] 3.449638
[1] 3.047247
[1] 3.047247
[1] 2.664583
[1] 2.738613
[1] 2.43975
[1] 4.035556
[1] 1.643168
[1] 2.880972
[1] 2.636737
[1] 3.701351
[1] 2.880972
[1] 3.779645
[1] 2.699206
[1] 2.073644
[1] 3.779645
[1] 3.866831
[1] 2.258318
[1] 3.047247
[1] 2.073644
[1] 2.370453
[1] 3.154739
[1] 1.974842
[1] 3.505098
[1] 4.157609
[1] 5.128353
[1] 3.359422
[1] 3.72827
[1] 1.870829
[1] 4.035556
[1] 3.449638
[1] 2.50998
[1] 3.016621
[1] 2.935821
[1] 2.073644
[1] 4.54082
[1] 3.271085
[1] 2.428992
[1] 2.880972
[1] 2.572751
[1] 5.468089
[1] 3.937004
[1] 3.909695
[1] 2.949576
[1] 2.664583
[1] 3.207135
[1] 3.258688
[1] 2.699206
[1] 3.457222
[1] 3.644957
[1] 4.076647
[1] 4.157609
[1] 3.779645
[1] 2.636737
[1] 3.047247
[1] 2.588436
[1] 3.701351
[1] 3.457222
[1] 3.735289
[1] 3.047247
[1] 2.949576
[1] 3.258688
[1] 3.505098
[1] 4.27785
[1] 3.55233
[1] 2.992053
[1] 2.819997
[1] 3.952094
[1] 3.420526
[1] 3.207135
[1] 2.878492
[1] 3.391165
[1] 3.457222
[1] 2.428992
[1] 3.309438
[1] 4.27785
[1] 2.428992
[1] 2.935821
[1] 3.690399
[1] 3.408672
[1] 3.866831
[1] 3.359422
[1] 3.644957
[1] 4.035556
[1] 3.505098
[1] 0.83666
[1] 6.363961
[1] 0.7071068
[1] 3.505098
[1] 3.016621
[1] 4.764452
[1] 3.271085
[1] 3.866831
[1] 3.146427
[1] 3.598942
[1] 3.331666
[1] 2.760262
[1] 3.781534
[1] 4.037326
[1] 3.690399
[1] 3.866831
[1] 3.146427
[1] 2.636737
[1] 4.035556
[1] 4.037326
[1] 3.55233
[1] 4.117327
[1] 3.994043
[1] 3.258688
[1] 2.43975
[1] 4.720775
[1] 3.408672
[1] 3.209361
[1] 3.016621
[1] 3.937004
[1] 4.117327
[1] 2.50998
[1] 4.549725
[1] 4.370355
[1] 4.764452
[1] 2.760262
[1] 2.167948
[1] 3.644957
[1] 3.391165
[1] 1.98806
[1] 3.937004
[1] 3.047247
[1] 3.457222
[1] 4.39155
[1] 3.735289
[1] 3.644957
[1] 3.207135
[1] 3.505098
[1] 2.760262
[1] 3.209361
[1] 3.209361
[1] 1.870829
[1] 2.070197
[1] 3.457222
[1] 3.937004
[1] 4.037326
[1] 3.781534
[1] 4.135215
[1] 2.636737
[1] 3.047247
[1] 4.503967
[1] 3.047247
[1] 2.880972
[1] 2.345208
[1] 3.598942
[1] 3.146427
[1] 4.32435
[1] 2.935821
[1] 3.937004
[1] 3.016621
[1] 2.636737
[1] 3.563706
[1] 2.280351
[1] 2.935821
[1] 3.619392
[1] 3.271085
[1] 4.035556
[1] 4.577377
[1] 3.359422
[1] 3.619392
[1] 4.41588
[1] 2.070197
[1] 3.082207
[1] 3.866831
[1] 3.114482
[1] 4.117327
[1] 3.101459
[1] 3.154739
[1] 4.076647
[1] 3.457222
[1] 3.154739
[1] 3.55233
[1] 1.870829
[1] 1.224745
[1] 2.12132
[1] 2.12132
[1] 9.192388
[1] 2.12132
[1] 9.192388
[1] 0.7071068
[1] 3.735289
[1] 2.992053
[1] 3.674235
[1] 3.598942
[1] 4.613644
[1] 2.992053
[1] 3.154739
[1] 2.992053
[1] 3.016621
[1] 4.157609
[1] 2.880972
[1] 2.572751
[1] 3.101459
[1] 3.258688
[1] 2.636737
[1] 2.992053
[1] 3.271085
[1] 3.834058
[1] 3.644957
[1] 3.258688
[1] 3.457222
[1] 2.664583
[1] 2.878492
[1] 2.345208
[1] 4.157609
[1] 3.779645
[1] 3.359422
[1] 2.507133
[1] 3.207135
[1] 4.32435
[1] 2.258318
[1] 3.598942
[1] 3.994043
[1] 3.309438
[1] 3.082207
[1] 3.391165
[1] 2.810694
[1] 4.117327
[1] 2.760262
[1] 3.505098
[1] 2.572751
[1] 4.27618
[1] 4.39155
[1] 2.370453
[1] 3.866831
[1] 3.258688
[1] 2.299068
[1] 3.359422
[1] 3.359422
[1] 3.047247
[1] 4.370355
[1] 3.271085
[1] 4.27618
[1] 3.309438
[1] 3.309438
[1] 3.994043
[1] 3.866831
[1] 3.154739
[1] 2.935821
[1] 3.55233
[1] 3.505098
[1] 2.992053
[1] 3.209361
[1] 3.016621
[1] 3.271085
[1] 3.507136
[1] 3.154739
[1] 0.5477226
[1] 2.12132
[1] 0.7071068
[1] 2.699206
[1] 3.674235
[1] 3.207135
[1] 2.819997
[1] 3.359422
[1] 4.929503
[1] 2.636737
[1] 4.505552
[1] 3.598942
[1] 2.43975
[1] 3.449638
[1] 2.572751
[1] 2.225395
[1] 2.949576
[1] 3.101459
[1] 4.929503
[1] 3.258688
[1] 2.588436
[1] 3.258688
[1] 3.779645
[1] 1.516575
[1] 3.207135
[1] 3.101459
[1] 3.082207
[1] 2.992053
[1] 5.8554
[1] 3.271085
[1] 3.408672
[1] 4.230839
[1] 2.878492
[1] 3.619392
[1] 2.149197
[1] 2.699206
[1] 3.207135
[1] 2.760262
[1] 3.563706
[1] 2.738613
[1] 2.428992
[1] 3.885872
[1] 4.636809
[1] 4.429339
[1] 2.949576
[1] 1.870829
[1] 3.952094
[1] 3.271085
[1] 3.507136
[1] 3.909695
[1] 3.457222
[1] 1.643168
[1] 3.735289
[1] 2.664583
[1] 3.644957
[1] 2.699206
[1] 2.810694
[1] 2.428992
[1] 4.157609
[1] 3.258688
[1] 3.359422
[1] 4.086563
[1] 2.760262
[1] 1.643168
[1] 3.082207
[1] 3.271085
[1] 1.788854
[1] 3.962323
[1] 3.082207
[1] 2.572751
[1] 4.549725
[1] 2.810694
[1] 3.047247
[1] 3.047247
[1] 3.016621
[1] 3.082207
[1] 3.391165
[1] 3.016621
[1] 3.408672
[1] 2.810694
[1] 4.460942
[1] 4.197505
[1] 1.378405
[1] 10.6066
[1] 4.949747
[1] 4.949747
[1] 3.535534
[1] 2.12132
[1] 6.363961
[1] 3.535534
[1] 7.778175
[1] 2.12132
[1] 4.949747
[1] 0.7071068
```

- here you can see that candidate randomisations are rejected until the standard deviation of units is below threshold (`SD` <=1.0) in order to approximate a uniform distribution across participants

practitioner_id <chr>	organisation_id <chr>	availability <chr>	times <chr>
e800013c0847	31615cf4ab8cf46473	monday	morning
4aa2133f826c	0c32d0b43bbe0eff84	saturday	evening
47a3a3095dec	0c32d0b43bbe0eff84	tuesday	evening
f117d25c0a66	0c32d0b43bbe0eff84	wednesday	morning
208d629694a7	0c32d0b43bbe0eff84	monday	morning
8e7dbe139a24	0c32d0b43bbe0eff84	thursday	morning
1e6dfd6976bb	0c32d0b43bbe0eff84	tuesday	morning
ebd07064549b	0c32d0b43bbe0eff84	wednesday	evening
0708281ef145	0c32d0b43bbe0eff84	saturday	morning
9431f62ac665	0c32d0b43bbe0eff84	saturday	morning

1-10 of 39 rows Previous 1 2 3 4 Next

bind assignments together for evaluation

Hide

```
library(data.table)

vec <- list.files(dirname(file.choose()),full.names=TRUE)
file_list <- c(vec[length(vec)],vec[-length(vec)])

d_list <- vector("list",length(file_list))
for(i in 1:length(file_list)) {
  z <- as.data.table(readr::read_csv(file_list[[i]],show_col_types = FALSE))
  z[, assignment := i]
  d_list[[i]] <- z
}

dt <- rbindlist(d_list,fill=TRUE)
dt
```

rowid <dbl>	practitioner_id <chr>	organisation_id <chr>	availability <chr>	times <chr>	assignment <int>
1	3bb9028368de	2311e69e2a2!61926d	monday	morning	1
2	0384a99a8532	8f*c79bad6b8b78fec	wednesday	morning	1
3	f426799c37a2	8f*c79bad6b8b78fec	sunday	evening	1
4	e58b05705de2	8f*c79bad6b8b78fec	tuesday	evening	1
5	9b1972bc35d6	8f*c79bad6b8b78fec	monday	morning	1
6	60d3a6d4dc64	8f*c79bad6b8b78fec	thursday	morning	1
7	8415055ffbcc	8f*c79bad6b8b78fec	thursday	morning	1
8	be023b4a683c	8f*c79bad6b8b78fec	wednesday	evening	1
9	83accb0f4b01	8f*c79bad6b8b78fec	thursday	evening	1
10	94d6671b327c	8f*c79bad6b8b78fec	wednesday	evening	1

1-10 of 156 rows Previous 1 2 3 4 5 6 ... 16 Next

Hide

```
# counts per day per period
as_tibble(dt) %>%
  group_by(assignment) %>%
  count(availability) %>%
  pivot_wider(names_from = assignment,values_from = n)
```

availability <chr>	1 <int>	2 <int>	3 <int>	4 <int>
monday	8	8	8	8
sunday	6	7	8	5
thursday	9	6	5	6
tuesday	8	7	7	7
wednesday	8	6	6	8
saturday	NA	5	5	5

6 rows

Hide

```
# descriptive statistics by day
as_tibble(dt) %>%
  group_by(assignment) %>%
  count(availability) %>%
  regroup(availability) %>%
  summarise(mean=mean(n),sd=sd(n))
```

availability <chr>	mean <dbl>	sd <dbl>
monday	8.00	0.000000
saturday	5.00	0.000000
sunday	6.50	1.290994
thursday	6.50	1.732051
tuesday	7.25	0.500000
wednesday	7.00	1.154701

6 rows

Bangladesh

blindly assign practitioner and organisation IDs using `ranid()` function

```
(bangladesh <- read_csv(file.choose(),show_col_types=FALSE) %>%
  rowid_to_column() %>%
  # blindly assign ranids to practitioners
  full_join(by="rowid",
    ranid(target_n = 15,
      dist_method = "lv",
      str_length = 12)) %>%
  rename("practitioner_id"=ranids) %>%
  group_by(organisation_name) %>%
  mutate(rowid = cur_group_id()) %>%
  # blindly assign ranids to organisations
  full_join(by="rowid",
    ranid(target_n = 4,
      max_symbols = 4,
      dist_method = "lv",
      str_length = 18)) %>%
  rename("organisation_id"=ranids) %>%
  ungroup() %->% tmp %>%
  select(organisation_id,practitioner_id,sunday:saturday))
```

organisation_id <chr>	practitioner_id <chr>	sunday <dbl>	monday <dbl>	tuesday <dbl>	wednesday <dbl>	thursday <dbl>	friday <dbl>	saturday <dbl>
dde2e2df3d2d18be9!	e7d967f0a19b	1	1	1	1	1	0	0
dde2e2df3d2d18be9!	da87eb25f209	1	1	1	1	1	0	0
dde2e2df3d2d18be9!	2a5ab70e5a80	1	1	1	1	1	0	0
dde2e2df3d2d18be9!	00034a6f7c45	1	1	1	1	1	0	0
dde2e2df3d2d18be9!	7a69ff13e503	1	1	1	1	1	0	0
dde2e2df3d2d18be9!	a1966071fcde	1	1	1	1	1	0	0
dde2e2df3d2d18be9!	a23a98acc596	1	1	1	1	1	0	0
5311e515104469554b	88edb5b0c00c	1	1	1	1	1	0	0
5311e515104469554b	bbd5bb366eab	1	1	1	1	1	0	0
5311e515104469554b	c80ca81343b1	1	1	1	1	1	0	0
5311e515104469554b	518df1056751	1	1	1	1	1	0	0
0915ac5527%2a84313	e8153ec46f92	1	1	1	1	1	0	0
0915ac5527%2a84313	4ef56f9aebff	1	1	1	1	1	0	0
5311e515104469554b	9ea5f7d96faf	1	1	1	1	1	0	0

1-14 of 15 rows — Previous 1 2 Next

```
# write_csv(tmp,"temp.csv")
rm(tmp)
```

make days-per-week availability matrix

```
(bangladesh_assignment <- bangladesh %>%
  group_by(practitioner_id) %>%
  mutate(sunday = case_when(sunday==1~"sunday",TRUE~NA_character_),
         monday = case_when(monday==1~"monday",TRUE~NA_character_),
         tuesday = case_when(tuesday==1~"tuesday",TRUE~NA_character_),
         wednesday = case_when(wednesday==1~"wednesday",TRUE~NA_character_),
         thursday = case_when(thursday==1~"thursday",TRUE~NA_character_),
         friday = case_when(friday==1~"friday",TRUE~NA_character_),
         saturday = case_when(saturday==1~"saturday",TRUE~NA_character_)) %>%
  unite("availability",sunday:saturday,na.rm=TRUE,remove=TRUE,sep=",") %>%
  mutate(availability = str_split(availability,pattern=",")))
```

organisation_id <chr>	practitioner_id <chr>	availability <list>
dde2e2df3d2d18be9!	e7d967f0a19b	<chr [5]>
dde2e2df3d2d18be9!	da87eb25f209	<chr [5]>
dde2e2df3d2d18be9!	2a5ab70e5a80	<chr [5]>
dde2e2df3d2d18be9!	00034a6f7c45	<chr [5]>
dde2e2df3d2d18be9!	7a69ff13e503	<chr [5]>
dde2e2df3d2d18be9!	a1966071fcde	<chr [5]>
dde2e2df3d2d18be9!	a23a98acc596	<chr [5]>
5311e515104469554b	88edb5b0c00c	<chr [5]>
5311e515104469554b	bbd5bb366eab	<chr [5]>
5311e515104469554b	c80ca81343b1	<chr [5]>
5311e515104469554b	518df1056751	<chr [5]>
0915ac5527%2a84313	e8153ec46f92	<chr [5]>
0915ac5527%2a84313	4ef56f9aebff	<chr [5]>
5311e515104469554b	9ea5f7d96faf	<chr [5]>

1-14 of 15 rows — Previous 1 2 Next

apply `counterbalance_across()` to days-per-week availability matrix

```
(bangladesh_assignment <- bangladesh_assignment[-15,] %>%
  regroup(practitioner_id,organisation_id,availability) %>%
  counterbalance_across(list_name = availability,print_runs = TRUE) %>%
  mutate(morning = 0,evening = 1) %>%
  unite("times",morning:evening,na.rm=TRUE,remove=TRUE,sep=",") %>%
  mutate(times = str_split(times,pattern=",")))
```

```
[1] 0.83666
```

practitioner_id <chr>	organisation_id <chr>	availability <chr>	times <list>
e7d967f0a19b	dde2e2df3d2d18be9!	sunday	<chr [2]>
da87eb25f209	dde2e2df3d2d18be9!	sunday	<chr [2]>
2a5ab70e5a80	dde2e2df3d2d18be9!	monday	<chr [2]>
00034a6f7c45	dde2e2df3d2d18be9!	thursday	<chr [2]>
7a69ff13e503	dde2e2df3d2d18be9!	tuesday	<chr [2]>
a1966071fcde	dde2e2df3d2d18be9!	thursday	<chr [2]>
a23a98acc596	dde2e2df3d2d18be9!	monday	<chr [2]>
88edb5b0c00c	5311e515104469554b	tuesday	<chr [2]>
bbd5bb366eab	5311e515104469554b	sunday	<chr [2]>
c80ca81343b1	5311e515104469554b	monday	<chr [2]>
518df1056751	5311e515104469554b	wednesday	<chr [2]>
e8153ec46f92	0915ac5527%2a84313	tuesday	<chr [2]>
4ef56f9aebff	0915ac5527%2a84313	monday	<chr [2]>
9ea5f7d96faf	5311e515104469554b	wednesday	<chr [2]>

1-14 of 14 rows

apply `counterbalance_across()` to times-per-day availability matrix

```
bangladesh_assignment %>%
  regroup(practitioner_id,organisation_id,availability) %>%
  counterbalance_across(list_name = times,print_runs = TRUE) %>%
  mutate(times = case_when(times==0~"morning",times==1~"evening")) %->% tmp
```

```
[1] 1.414214
[1] 4.242641
[1] 0
```

practitioner_id <chr>	organisation_id <chr>	availability <chr>	times <chr>
e7d967f0a19b	dde2e2df3d2d18be9!	sunday	evening
da87eb25f209	dde2e2df3d2d18be9!	sunday	evening
2a5ab70e5a80	dde2e2df3d2d18be9!	monday	morning
00034a6f7c45	dde2e2df3d2d18be9!	thursday	morning
7a69ff13e503	dde2e2df3d2d18be9!	tuesday	evening
a1966071fcde	dde2e2df3d2d18be9!	thursday	morning
a23a98acc596	dde2e2df3d2d18be9!	monday	evening
88edb5b0c00c	5311e515104469554b	tuesday	evening
bbd5bb366eab	5311e515104469554b	sunday	morning
c80ca81343b1	5311e515104469554b	monday	evening
518df1056751	5311e515104469554b	wednesday	morning
e8153ec46f92	0915ac5527%2a84313	tuesday	morning
4ef56f9aebff	0915ac5527%2a84313	monday	evening
9ea5f7d96faf	5311e515104469554b	wednesday	morning

1-14 of 14 rows

```
  # %>% write_csv("Bangladesh_Beneficiary1.csv")
```

batch beneficiary interview assignment sheets /not run/

````
```r
input <- read_csv(file.choose(),show_col_types = FALSE)

input %<>%
  group_by(practitioner_id) %>%
  mutate(sunday = case_when(sunday==1~\sunday\,TRUE~NA_character_),
         monday = case_when(monday==1~\monday\,TRUE~NA_character_),
         tuesday = case_when(tuesday==1~\tuesday\,TRUE~NA_character_),
         wednesday = case_when(wednesday==1~\wednesday\,TRUE~NA_character_),
         thursday = case_when(thursday==1~\thursday\,TRUE~NA_character_),
         friday = case_when(friday==1~\friday\,TRUE~NA_character_),
         saturday = case_when(saturday==1~\saturday\,TRUE~NA_character_)) %>%
  unite(\availability\,sunday:saturday,na.rm=TRUE,remove=TRUE,sep=\
````

Reproducibility Information

```
sessionInfo()
```

```
R version 4.2.1 (2022-06-23)
Platform: x86_64-apple-darwin17.0 (64-bit)
Running under: macOS Catalina 10.15.7

Matrix products: default
BLAS:   /System/Library/Frameworks/Accelerate.framework/Versions/A/Frameworks/vecLib.framework/Versions/A/libBLAS
.dylib
LAPACK: /Library/Frameworks/R.framework/Versions/4.2/Resources/lib/libRlapack.dylib

locale:
[1] en_AU.UTF-8/en_AU.UTF-8/en_AU.UTF-8/C/en_AU.UTF-8/en_AU.UTF-8

attached base packages:
[1] parallel  stats     graphics  grDevices utils     datasets  methods   base

other attached packages:
 [1] data.table_1.14.6 doParallel_1.0.17 iterators_1.0.14  foreach_1.5.2     c4c_0.0.9         quanteda_3.2.3
 [7] stringdist_0.9.8  ids_1.0.1         louisdesu_0.0.1   forcats_0.5.2     stringr_1.4.1     dplyr_1.0.10
[13] purrr_0.3.5       readr_2.1.2       tidyr_1.2.1       tibble_3.2.1      ggplot2_3.4.0     tidyverse_1.3.2
[19] designr_0.1.12

loaded via a namespace (and not attached):
 [1] httr_1.4.6          bit64_4.0.5         vroom_1.5.7         jsonlite_1.8.4      splines_4.2.1
 [6] modelr_0.1.9        RcppParallel_5.1.5  assertthat_0.2.1    googlesheets4_1.0.1 cellranger_1.1.0
[11] pillar_1.9.0        backports_1.4.1     lattice_0.20-45     glue_1.6.2          uuid_1.1-0
[16] rvest_1.0.3         minqa_1.2.4         colorspace_2.0-3    Matrix_1.5-1        pkgconfig_2.0.3
[21] broom_1.0.1         haven_2.5.1         scales_1.2.1        tzdb_0.3.0          lme4_1.1-30
[26] timechange_0.1.1    googledrive_2.0.0   generics_0.1.3      ellipsis_0.3.2      pacman_0.5.1
[31] withr_2.5.0         cli_3.6.1           magrittr_2.0.3      crayon_1.5.2        readxl_1.4.1
[36] stopwords_2.3       fs_1.5.2            fansi_1.0.4         nlme_3.1-159        MASS_7.3-58.1
[41] xml2_1.3.3          tools_4.2.1         hms_1.1.3           gargle_1.2.1        lifecycle_1.0.3
[46] munsell_0.5.0       reprex_2.0.2        compiler_4.2.1      rlang_1.1.1         grid_4.2.1
[51] nloptr_2.0.3        rstudioapi_0.14     boot_1.3-28         codetools_0.2-18    gtable_0.3.1
[56] DBI_1.1.3           R6_2.5.1            lubridate_1.9.0     knitr_1.40          bit_4.0.4
[61] utf8_1.2.3          fastmatch_1.1-3     stringi_1.7.8       Rcpp_1.0.9          vctrs_0.6.2
[66] dbplyr_2.2.1        tidyselect_1.2.0    xfun_0.33
```

```
userSessionInfo()
```

```
User-defined functions:
No user-defined functions found.
```


- please direct any technical correspondence to Louis Klein : `elrha-c4c@louisdesu.com`

1. Date created: 04-04-22↩
```
